# Detecting and dissecting signaling crosstalk via the multilayer network integration of signaling and regulatory interactions

**DOI:** 10.1093/nar/gkad1035

**Published:** 2023-11-11

**Authors:** Leonardo Martini, Seung Han Baek, Ian Lo, Benjamin A Raby, Edwin K Silverman, Scott T Weiss, Kimberly Glass, Arda Halu

**Affiliations:** Channing Division of Network Medicine, Department of Medicine, Brigham and Women’s Hospital, Harvard Medical School, Boston, MA, 02115, USA; Department of Computer, Control, and Management Engineering, Sapienza University of Rome, Rome, 00185, Italy; Division of Pulmonary Medicine, Boston Children’s Hospital, Harvard Medical School, Boston, MA, 02115, USA; Department of Biostatistics, Harvard T.H. Chan School of Public Health, Boston, MA, 02115, USA; Division of Pulmonary Medicine, Boston Children’s Hospital, Harvard Medical School, Boston, MA, 02115, USA; Channing Division of Network Medicine, Department of Medicine, Brigham and Women’s Hospital, Harvard Medical School, Boston, MA, 02115, USA; Channing Division of Network Medicine, Department of Medicine, Brigham and Women’s Hospital, Harvard Medical School, Boston, MA, 02115, USA; Channing Division of Network Medicine, Department of Medicine, Brigham and Women’s Hospital, Harvard Medical School, Boston, MA, 02115, USA; Department of Biostatistics, Harvard T.H. Chan School of Public Health, Boston, MA, 02115, USA; Channing Division of Network Medicine, Department of Medicine, Brigham and Women’s Hospital, Harvard Medical School, Boston, MA, 02115, USA

## Abstract

The versatility of cellular response arises from the communication, or crosstalk, of signaling pathways in a complex network of signaling and transcriptional regulatory interactions. Understanding the various mechanisms underlying crosstalk on a global scale requires untargeted computational approaches. We present a network-based statistical approach, MuXTalk, that uses high-dimensional edges called multilinks to model the unique ways in which signaling and regulatory interactions can interface. We demonstrate that the signaling-regulatory interface is located primarily in the intermediary region between signaling pathways where crosstalk occurs, and that multilinks can differentiate between distinct signaling-transcriptional mechanisms. Using statistically over-represented multilinks as proxies of crosstalk, we infer crosstalk among 60 signaling pathways, expanding currently available crosstalk databases by more than five-fold. MuXTalk surpasses existing methods in terms of model performance metrics, identifies additions to manual curation efforts, and pinpoints potential mediators of crosstalk. Moreover, it accommodates the inherent context-dependence of crosstalk, allowing future applications to cell type- and disease-specific crosstalk.

## Graphical Abstract

**Graphical Abstract:**
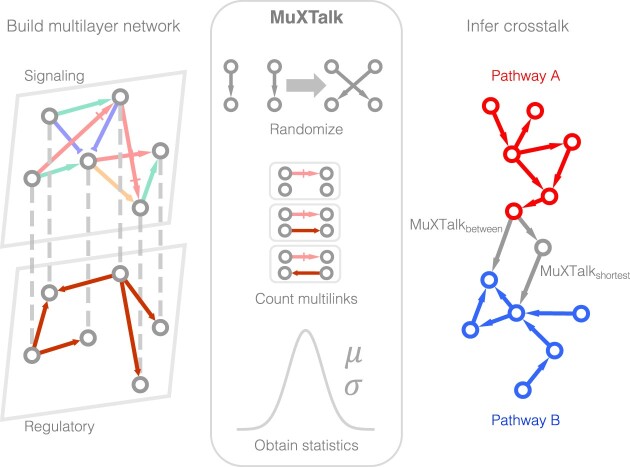

## Introduction

Signal transduction pathways are sequences of biomolecular interactions through which cells respond to their environment, processing internal and external stimuli through a succession of receptors, intermediate signaling proteins, transcription factors (TFs) and their target genes. Signaling pathways do not perform their functions in isolation; they instead operate within a global network of protein–protein interactions (1,2). Due to the tightly knit and often overlapping way in which signaling pathways are embedded in this global network, stimuli received by one pathway frequently lead to downstream effects in another pathway, resulting in what is called signaling crosstalk. Signaling crosstalk helps to combine the relatively few canonical signaling pathways in different ways, giving rise to the vast range of cellular responses observed in healthy development and homeostasis, as well as in disease (3,4).

Analyzing signaling networks presents numerous challenges. First, signaling networks consist of multiple types of edges, i.e. signaling interactions. Some types of signaling interactions number in the thousands, whereas other types include only a handful. Second, signaling edges are highly context-dependent: An edge between the same pair of nodes may represent different relationships (e.g. inhibition versus activation) in different pathways. Third, signaling is inherently entangled with transcriptional regulation since TFs and their targets form the last stage of signaling cascades. Indeed, the transcriptional control of a pathway by another pathway has been shown to be an important contributing mechanism to crosstalk (5–7).

The computational modeling of signaling crosstalk has traditionally focused on ‘critical’ nodes, i.e. the common genes between pathways, that were deemed to serve as a junction to cause crosstalk (8,9). This idea has later been extended to gene sets, inferring crosstalk by the similarity between enriched functional annotation terms (10). Network methods have added more sophistication to these overlap- and enrichment-based approaches by defining crosstalk based on the interactions that lie between signaling pathways (11–15). Recently, further refined network-based approaches that consider the canonical definition of signal transduction have emerged (16,17). These methods model crosstalk as the propagation of signals in an underlying network from receptors to TFs and consider long-range interactions between signaling pathways.

Despite the utility and individual advantages of each of these approaches, each has one or more of the following limitations that impact their specificity, predictive power and context-specific application: (i) Treating signaling pathways as gene sets and disregarding the interactions of individual genes within and between them; (ii) Not differentiating between the multiple types of signaling interactions; (iii) Focusing solely on the set of receptors and TFs in a pathway as the input and not the remaining proteins in the pathway; (iv) Not modeling additional crosstalk mechanisms such as crosstalk by PPIs, feedback loops, and downstream TF targets that are members of other pathways; (v) Relying on absolute numbers of edges without providing a statistical background; (vi) Being limited in scope, typically restricting their analyses to a handful of the most well-described signaling pathways.

In this study, we posit that a deeper understanding of cellular signaling, including crosstalk, requires a unified view of signal transduction and transcriptional regulatory events in the cell (18,19). To integrate cell signaling and gene regulation while tackling the above-mentioned challenges around analyzing signaling networks, we use multilayer networks (20,21), which can simultaneously keep track of multiple types of concurrent and context-dependent edges in the form of distinct layers of networks. In particular, we use high-dimensional edges called *multilinks* (22,23) to model the unique ways in which signaling and regulatory interactions can interface with each other. We introduce a computational framework, MuXTalk, that exploits the statistics of multilinks to (i) characterize signaling pathways, (ii) infer signaling crosstalk, specifically by searching for statistically significant regulatory edges connecting pairs of signaling pathways, and (iii) study the mechanisms and potential molecular conduits contributing to signaling crosstalk. In our benchmarks, MuXTalk performs better than gene set-based and other network-based approaches in identifying crosstalk. We find recent literature evidence of crosstalk previously not captured in extensive curation efforts, signifying additions to current benchmarks. Crosstalk inferences in our ‘discovery’ set of pathway pairs are supported in the literature. Overall, MuXTalk addresses the limitations outlined above by being a network-based, statistical approach that can accommodate context specificity in the biomolecular interactions at the signaling and transcriptional regulation levels and model multiple crosstalk mechanisms. Covering 60 KEGG signaling pathways, it expands the search space of the currently available crosstalk databases with a more than five-fold increase of potentially crosstalking pathway pairs. The MuXTalk package and web app are available at (https://github.com/r-duh/MuXTalk).

## Materials and methods

### Building the signaling layer

We curated a list of 60 cellular signaling pathways from KEGG and downloaded their schemas as KGML (KEGG Markup Language) files in May and October 2020 (see Supplementary Table S1 for a list of pathways). We parsed each KGML file to build a directed graph consisting of KEGG ‘entries’ (nodes) and ‘relations’ (edges). Since KEGG relations include both directed and undirected interaction types, we represented undirected interactions as bidirectional edges (see the Materials and methods section on multilinks for details). Furthermore, in KEGG pathway maps, composite objects such as protein complexes are often represented as a single entry. We disaggregated such gene groups and protein complexes into their individual genes and represented these gene groups as complete graphs in which every constituent gene was connected to every other gene in the group. To disambiguate these interconnecting edges within gene groups, we separately annotated them as ‘within_group’ edges, in addition to the existing KEGG relations. We aggregated the 60 KEGG graphs into a directed heterogeneous multigraph where each directed edge is annotated with the interaction type as well as the signaling pathway it belongs to, allowing for multiple edges between the same pair of nodes. Overall, this precursor KEGG network had 3306 nodes and 44 286 edges spanning 4 node types (gene, compound, map and ortholog) and 13 interaction types (activation, binding/association, compound, dephosphorylation, dissociation, expression, indirect effect, inhibition, phosphorylation, repression, state change, ubiquitination, within group). To build the overall signaling layer, we combined the KEGG signaling network with the large-scale human protein-protein interaction (PPI) network curated by Cheng *et al.* (24). We represented PPI edges as bidirectional and annotated them separately with the ‘ppi’ edge type. During the merging of the KEGG and PPI networks, if a known signaling interaction from the KEGG network coincided with a PPI edge, the KEGG signaling edge superseded the PPI edge. As part of our quality control procedure, the few KEGG edges with unknown interaction type, as well as the self-loops in the PPI and KEGG networks, were removed from the signaling layer. Finally, we removed the ‘GErel’ type of edges (‘expression’ and ‘repression’) from the signaling layer to prevent redundancy with the gene regulatory network layer. After these refinements, the final KEGG signaling network consisted of 2363 genes and 40 966 edges (24 222 unique edges) spanning 60 signaling pathways and 11 types of signaling interactions. For our sensitivity analyses, we used the STRING database (https://string-db.org/) and the GRNdb (http://www.grndb.com/). Both of these databases provide weighted networks that have confidence scores as edge weights, which allowed us to perform sensitivity analyses at multiple confidence thresholds and explore PPIs and GRNs at various network sizes and densities. For the STRING database, we built PPI networks at two confidence score (CS) cutoffs (700 and 900); for the GRNdb, we built GRNs at two normalized enrichment score (NES) cutoffs (5) and (7). We built the GRNdb-based network by taking the union of tissue-specific GTEx edges in the GRNdb database.

### Building the signaling-regulatory multilayer network

As the basis of our multilink-based crosstalk inference framework, we built a multilayer network consisting of a signaling layer and a gene regulatory layer. As the gene regulatory layer, we used the human gene regulatory network (GRN) previously described in (25). Briefly, the human GRN was constructed in (25) by scanning the entire hg19 genome for 695 human TF motifs, and a TF was connected to a gene if the motif hit for that TF was in the promoter region of that gene. We considered in our analyses GRNs created using three motif scan p-value thresholds (*P*< 1 × 10^−4^, *P*< 1 × 10^−5^ and *P*< 1 × 10^−6^) corresponding to a wide range of network densities (Supplementary Table S2). Since our multilayer network is technically a multiplex network in which the complete set of nodes is present in all layers, even if as isolated nodes, we only retained genes and their interactions in the signaling network, discarding other KEGG entries such as ‘maps,’ ‘orthologs’ or ‘compounds’. We then pruned the GRN to only include the genes present in the signaling layer. The number of TFs, targets, edges and network densities post-pruning are shown in Supplementary Table S2. We used all three versions of GRNs in our sensitivity analyses (Supplementary Figures S2–7 and 9) and used the *P* < 1 × 10^−6^ cutoff GRN in the rest of the analyses throughout the manuscript. For memory efficiency in downstream calculations, we stored each layer as a sparse matrix. Furthermore, due to the multigraph nature of the signaling layer, where more than one type of edge can exist between the same pair of nodes, we stored each signaling interaction type $e$ as a separate sparse matrix. For a larger rewiring space, we added PPI edges to each interaction-specific KEGG layer (named ‘KEGG_e’ layers). In addition, to avoid multiple-counting of the $( {0,\ R} )$ type of multilinks (detailed in the following section), we collapsed all KEGG interaction types and PPIs on a single network separately (named the ‘KEGGPPI’ layer). KEGGPPI thus represents all the interactions on the signaling layer in a collapsed form without differentiating between the individual types of signaling interactions or PPIs. Altogether, this resulted in sparse matrices for the following layers: 1 for the GRN layer, 11 for the interaction-specific KEGG_e layers, and 1 for the collapsed KEGGPPI layer (Supplementary Figure S13).

### Counting multilinks and obtaining their statistics

Our crosstalk inference method relies on the statistics of multilinks, which are higher order edges that can represent, in a compact way, unique combinations of interactions between the signaling layer and the regulatory layer. Hence, multilinks are a means to keep track of which kind of signaling interaction coincides with a gene regulatory event, and whether these interactions occur in the same or opposite direction. In this context, only one signaling interaction type is considered at a time with the corresponding regulatory edge. As a shorthand for denoting multilinks, we represent signaling interactions by integers $S$ in the interval $[ {0,\ 11} ]$, and regulatory interactions by the integers $R$ in $\{ { - 1,\ 0,\ 1} \}$ to account for directionality, where 0 denotes no edge. Together, this resulted in $12\ \times \ 3 = 36$ multilink types denoted by the pair of integers $( {S,\ R} )$. We extracted the number of each multilink type by counting all instances of a given $( {S,\ R} )$ pair across the tensor formed by the stacked adjacency matrices of the multilayer network. To be able to differentiate between signaling and regulatory edges that are in the same and opposite directions, we filled the symmetric position of a given directed edge with its negative value in the S and R adjacency matrices. This procedure enabled us to capture all overlapping signaling and regulatory edges in both directions at once. Once the signaling and regulatory layers’ sparse matrices were stacked and reshaped by our algorithm, equivalent representations of the same multilink type (e.g. (1, - (1) and (-1, 1)) were collapsed onto one representative multilink type. For the undirected edges, we filled both symmetric positions with the same positive value to ensure that the counts of the equivalent representations of multilinks that involve undirected signaling edges (e.g. (2,1) and (2, - (1)) were exactly equal. Finally, we corrected for the double-counting of undirected signaling edges not overlapping with any regulatory edges (e.g. (2,0)). We note that, while we took care to avoid double counting of the undirected edge types for completeness, the scale invariance of z-scores under linear transformations additionally guarantees that the statistics (z-scores and *P*-values) for each multilink type and, consequently, MuXTalk scores, are not biased by this record-keeping procedure. Since counting multilinks quickly becomes computationally burdensome for large multidimensional arrays such as ours $( {2\ \times 16080 \times 16080} )$, we introduced a custom hash to represent each of the 36 multilink types as a unique number. To correctly account for the concurrent edges in the signaling network, we performed the counting step separately on the multilayer formed by the sparse matrix of each signaling interaction type $S$ (Supplementary Figure S13). To prevent multiple-counting, we counted the $( {0,\ R} )$ type of multilinks, i.e. cases where no edge is present in the signaling layer, on the multilayer network formed by the KEGGPPI layer and the gene regulatory layer.

To assess whether a given multilink count is statistically more or less than what would be expected by chance, we generated ensembles of randomized networks. To counter degree bias, we used a degree-preserving randomization method that relies on the random pairwise rewiring of edges (26). To break potential existing degree correlations between layers, we performed this randomization procedure for each layer separately. We generated and stored 500 randomized networks for each of the 13 layers described above, resulting in 250 000 unique randomized multilayer networks for each signaling type-GRN layer pair. To quantify the extent of over- or under-representation of a given multilink count and its statistical significance, we calculated z-scores and two-tailed empirical *P*-values, respectively, based on the actual ${c}_a$ and random counts ${c}_r$ such that


\begin{eqnarray*}z = \frac{{{c}_a - \left\langle {{c}_r} \right\rangle }}{{{\sigma }_{{c}_r}}}\end{eqnarray*}

and


\begin{eqnarray*}{p}_{emp.} = \left\{ {\begin{array}{@{}*{1}{c}@{}} {\frac{{P\left( {{c}_r \ge {c}_a} \right)}}{{{N}_{rand}}}\ if\ z >0}\\ {\frac{{P\left( {{c}_r \le {c}_a} \right)}}{{{N}_{rand}}}\ if\ z \,<\, 0} \end{array}} \right.\end{eqnarray*}

where $\langle {{c}_r} \rangle$ and ${\sigma }_{{c}_r}$ are the mean and standard deviation of ${c}_r$ and ${N}_{rand}$ is the number of random instances. In our analyses, we used ${N}_{rand} = 100$, but this parameter is adjustable by the user. For the reproducibility of our results, the randomized layers used in the benchmarks were drawn in a deterministic manner from this precomputed ensemble (see Supplementary Table Sii3 for the allocation schema of randomized networks).

### Using multilink statistics to infer crosstalk

In our multilink-based crosstalk inference framework, we used multilinks of type $( {S,\ 1} )$ and $( {S,\ - 1} )$ as proxies of crosstalk and deemed as crosstalking the pairs of pathways for which at least one of these multilink types is significantly over-represented (${p}_{emp.} \le 0.05,\ z >0$). We then devised two approaches to model signaling crosstalk based on multilink statistics: (i) based on the direct edges between a pair of signaling pathways (‘MuXTalk_between_’) and (ii) based on the shortest paths between a pair of signaling pathways (‘MuXTalk_shortest_’) in the signaling layer (‘KEGGPPI’).

For MuXTalk_between_, multilink statistics were obtained for the edges connecting Pathway A and Pathway B such that all edges directly connecting the genes in the two pathways were accounted for, excluding (i) the edges within pathways and (ii) the edges connecting the nodes that are common to both pathways (Figure 3B). These criteria were to ensure that only the edges directly between the two pathways were counted. Effectively, we did this by slicing the adjacency matrices of each layer by the mutually exclusive set of nodes in each pathway. We calculated multilink statistics on this final subset of the adjacency tensor.

For MuXTalk_shortest_, multilink statistics were obtained for the edges belonging to the shortest paths connecting Pathway A and B, following the steps below:

(i) We determined the mutually exclusive sets of nodes between signaling Pathway A and B.

(ii) For each pair of nodes in this mutually exclusive set of nodes, we computed the shortest path, when such a path exists.

(iii) We excluded from these shortest paths any node that belongs to Pathway A or B to identify the set of ‘intermediary nodes’ that exclude the ‘within-pathway’ edges. To fine-tune the reach of the shortest paths between pathways, we introduced a shortest path (sp) threshold that controls the number of intermediary nodes between pairs of pathways. In our simulations, we used sp threshold values of 1, 2 and ‘no threshold’, meaning that shortest paths of any length were considered.

(iv) Using the intermediary nodes, we identified the intermediary shortest paths connecting each pair of mutually exclusive nodes in Pathway A and B.

(v) We aggregated all edges in intermediary shortest paths for all sets of mutually exclusive nodes in Pathway A and B. We calculated multilink statistics on this final set of edges.

In both MuXTalk_between_ and MuXTalk_shortest_, we performed the same operations on the randomized multilayer network ensembles and calculated the z-scores and empirical *P*-values for each multilink type, as described in the previous section. Using these statistics, we then ranked signaling pathway pairs by their (i) number of significantly over-represented (${p}_{emp.} \le 0.05,\ z >0$) multilink types, (ii) lowest *P*-value, and (iii) highest z-score. The signaling pathway pairs with the highest number of significantly over-represented multilinks of type $( {S,\ 1} )$ and $( {S,\ - 1} )$, with the lowest empirical *P*-values, and with the highest z-scores were thus prioritized as the most likely pathway pairs to crosstalk. To reflect this ranking, we calculated the MuXTalk score as:


\begin{eqnarray*}&& MuXTalk\ score \\ &&= \left\{ {\begin{array}{@{}*{1}{c}@{}} {1000{n}_{sig} - \ lo{g}_{10}\left( {{{\tilde{p}}}_{emp.} + 0.001} \right)*\tilde{z}\ if\ {\sigma }_{{c}_r} \ne 0\ }\\ {1000{n}_{sig} - \ lo{g}_{10}\left( {{{\tilde{p}}}_{emp.} + 0.001} \right)\ if\ {\sigma }_{{c}_r} = 0\ } \end{array}} \right.\end{eqnarray*}

where ${n}_{sig}$ is the number of significant multilink types and ${\tilde{p}}_{emp.}$ and $\tilde{z}$ are the best empirical p-values and z-scores, respectively.

### Benchmarking

To assess the performance of MuXTalk to infer crosstalk as compared to other methods, we devised a benchmark that uses the literature-curated crosstalking pathways available in the XTalkDB database (27). XTalkDB provides an exhaustive survey of the literature (up to the year 2017) to identify both the presence and the absence of crosstalk between all pairs of the considered pathways, resulting in what we can use as true positives and true negatives to generate Receiver Operating Characteristic (ROC) and Precision-Recall (PR) curves. Thus, we use the term ‘performance’ to refer to model performance as measured by area under the ROC and PR curves based on the true positive and true negative cross-talking pathway pairs as defined by XTalkDB. Of the 26 KEGG signaling pathways in XTalkDB, 25 were within the list of 60 KEGG pathways we used to build the signaling layer. Of the 600 possible ordered pairs in these 25 pathways, 331 were marked as crosstalking in XTalkDB.

We compared MuXTalk with node and edge overlap (8,9), direct edges (13,28) and shortest paths (16) between signaling pathways. We detail the implementation of each method below:

*Node overlap between signaling pathways:* We performed a two-tailed Fisher Exact test to assess the significance of overlap between the nodes of Pathway A and Pathway B given the number of nodes in each pathway and the total number of genes in the 60 KEGG pathways considered. We adjusted the Fisher’s Exact *P*-values for multiple testing using the Benjamini-Hochberg procedure. We then ranked pathway pairs based on the false discovery rate (FDR) values, with the lowest FDR pathway pair ranking highest as the most likely to crosstalk.

*Edge overlap between signaling pathways:* Similar to node overlap, we performed a two-tailed Fisher’s Exact test to assess the significance of overlap between the edges of Pathway A and Pathway B given the number of edges in each pathway and the total number of possible edges in the 60 KEGG pathways considered. We adjusted the Fisher’s Exact *P*-values for multiple testing using the Benjamini-Hochberg procedure. We ranked pathway pairs based on the false discovery rate (FDR) values, with the lowest FDR pathway pair ranking highest as the most likely to crosstalk.

*Direct edges between signaling pathways:* We used direct interactions between pairs of pathways as proxies of signaling crosstalk, as done previously by Korcsmaros *et al.* (13). For each pathway pair, we determined the number of direct interactions between them and compared this value to the one obtained from randomized versions of the KEGG signaling network to calculate z-scores and empirical *P*-values, as described in the previous section. We finally ranked pathway pairs by their empirical *P*-values and z-scores, prioritizing pairs with the lowest p-values and highest z-scores as the most likely to crosstalk.

*Shortest paths between signaling pathways (Crosstalk Statistic* $\chi$ *)*. Following the procedure in (16), we used the shortest paths between the membrane receptor proteins (receptors) and the transcription factors (TFs) in each KEGG signaling pathway to calculate the crosstalk statistic $\chi$. The receptor and TF data were obtained from Almen *et al.* (29) and Lambert *et al.* (30), respectively. For the receptor data, we parsed gene symbols from International Protein Index (IPI) Descriptions. We converted both sets of gene symbols to Entrez IDs. We extracted *K* shortest paths between receptors and TFs using Yen’s algorithm (31) to calculate the crosstalk statistic $\chi$ as described in (16) for *K* values between 1 and 100. We repeated the $\chi$ calculation on randomized networks to obtain z-scores and empirical *P*-values. We then ranked pathway pairs by their empirical *P*-values and z-scores, prioritizing pairs with the lowest p-values and highest z-scores as the most likely to crosstalk.

To compare the model performance of the above methods, we used their respective ranked list of pathway pairs to generate Receiver Operating Characteristic (ROC) and Precision-Recall (PR) curves, and calculated the area under these curves. Since all methods have instances where the magnitude of the respective crosstalk metric for a pair of pathways cannot be determined (e.g. due to the absence of node or edge overlap between pathways, the absence of TFs or receptors in a given pathway or shortest paths connecting them, the lack of statistically over-represented multilink types), we adopted two strategies, one deterministic and one stochastic, when generating the ROC and PR curves. For the deterministic assessment, we only used the pathway pairs that were ‘detected’ (i.e. pathway pairs that were assigned crosstalk statistics and corresponding *P*-values and z-scores and that could hence be ranked) in the ROC and PR curves. For the stochastic assessment, we used all 600 pathway pairs in the benchmark, including the ones that did not have crosstalk metrics, and shuffled the ranks of such pairs 1000 times. This procedure resulted in ROC and PR curves that were ‘fuzzy’ after the detection threshold, and the area under the curves (AUCs) (Supplementary Figure S14) for the stochastic approach were represented by the mean and standard deviation of all shuffled AUCs.

*Leave-one-layer-out cross-validation*. To assess the influence of each signaling interaction type on the overall crosstalk inference performance, we designed a leave-one-layer-out cross-validation analysis in which we excluded the significant multilinks associated with the left-out layer when prioritizing potentially crosstalking pathways. We note that we did not include the PPI layer in our leave-one-layer-out analysis due to its crucial role in connecting the signaling pathways in MuXTalk_shortest_.

### Using PubMed query-guided literature curation to assess the inferences in the discovery set of pathways

Our complete set of 60 KEGG signaling pathways encompasses 3540 ordered pathway pairs whose crosstalk can be explored. We call the pathway pairs outside of the 600 benchmark pairs our ‘discovery’ set. Since there are currently no literature-curated databases on crosstalk among this larger set, we devised a complementary approach that utilizes a combination of PubMed queries and manual curation to look for evidence of crosstalk in the discovery set. We first manually curated a list of keywords for each signaling pathway (Supplementary Table S6). We then performed automated PubMed searches using the rentrez R package (32), which leverages the Medical Subject Heading (MeSH) term search functionality of PubMed for a controlled vocabulary of medical terms, allowing for complex and ontologically coherent queries. We followed an approach similar to (27) when constructing our queries, using the general form ‘(Pathway A) AND (Pathway B) AND (signaling) AND (crosstalk)’. An example query translation for the crosstalk between the TGF-beta and the cGMP-PKG signaling pathways is as follows: ‘((\’transforming growth factor beta\‘[MeSH Terms] OR (\’transforming\‘[All Fields] AND \’growth\‘[All Fields] AND \’factor\‘[All Fields] AND \’beta\‘[All Fields]) OR \’transforming growth factor beta\‘[All Fields] OR (\’tgf\‘[All Fields] AND \’beta\‘[All Fields]) OR \’tgf beta\‘[All Fields]) OR TGFB[All Fields] OR \’Transforming growth factor\‘[All Fields]) AND (cGMP[All Fields] OR PKG[All Fields] OR cGMP-PKG[All Fields]) AND (\’signal transduction\‘[MeSH Terms] OR (\’signal\‘[All Fields] AND \’transduction\‘[All Fields]) OR \’signal transduction\‘[All Fields] OR \’signaling\‘[All Fields]) AND ((\’cross reactions\‘[MeSH Terms] OR (\’cross\‘[All Fields] AND \’reactions\‘[All Fields]) OR \’cross reactions\‘[All Fields] OR \’crosstalk\"[All Fields]) OR cross-talk[All Fields]) AND (1900[PDAT] : 2022[PDAT])’. We considered each query that resulted in at least one publication for the given pathway pair as a potential positive and created precision-rank plots for the top-ranked pathway pairs to track MuXTalk’s rate of capturing these potential positives. However, since the presence of the ‘crosstalk’ keyword and its synonyms is not a guarantee of crosstalk occurring between the given two pathways, we performed an additional manual curation approach on the PubMed query results for the top 50 pathway pairs inferred by MuXTalk. Briefly, (i) we used the PubMed IDs (PMIDs) returned by our PubMed queries to identify the potential PMIDs to be screened; (ii) we screened first the title, then the abstract, and finally the main text of an identified paper for sentences that support crosstalk. If we found evidence of crosstalk by this method, we marked this pair as From_PubMed_Query = Yes and then moved to the next pair. We noted the direction of the crosstalk as inferred from these key sentences wherever possible; (iii) For cases in which none of the PMIDs returned by the PubMed query had any crosstalk evidence, we performed our own web browser search manually to look for crosstalk between the given pathways. If we found evidence of crosstalk by this method, we marked this pair as From_PubMed_Query = No. We noted the direction of the crosstalk as inferred from these key sentences wherever possible. For each pathway pair, we recorded the PMID of the article, the key sentence(s) that imply signaling crosstalk, the direction of crosstalk (a→b or b→a, with only a→b being counted as a ‘positive’), and any additional notes of importance. The results of our manual curation are provided in Supplementary Files S1 and 2.

### Using ChIP-seq data for additional support of MuXTalk inferences

We used data from ChIP-Atlas (https://chip-atlas.org/) to identify the potential transcriptional regulators of TGF-β signaling pathway by measuring the enrichment of ChIP-seq peaks corresponding to TF regulatory elements. We utilized the Enrichment Analysis functionality of ChIP-Atlas with the following parameters for human genome build hg19: Experiment type: ‘ChIP: TFs and others’; Cell type class: All; Threshold for significance: 50, Distance range from TSS: -50 bp to + 50 bp. We used the genes in the TGF-β signaling pathway as the input gene-set and all the genes in the KEGG signaling network comprised of 60 KEGG signaling paths (excluding the TGF-β signaling pathway genes) as the background against which peak enrichment is calculated.

## Results

### The multilayer network of signaling and regulatory interactions

**Figure 1.**
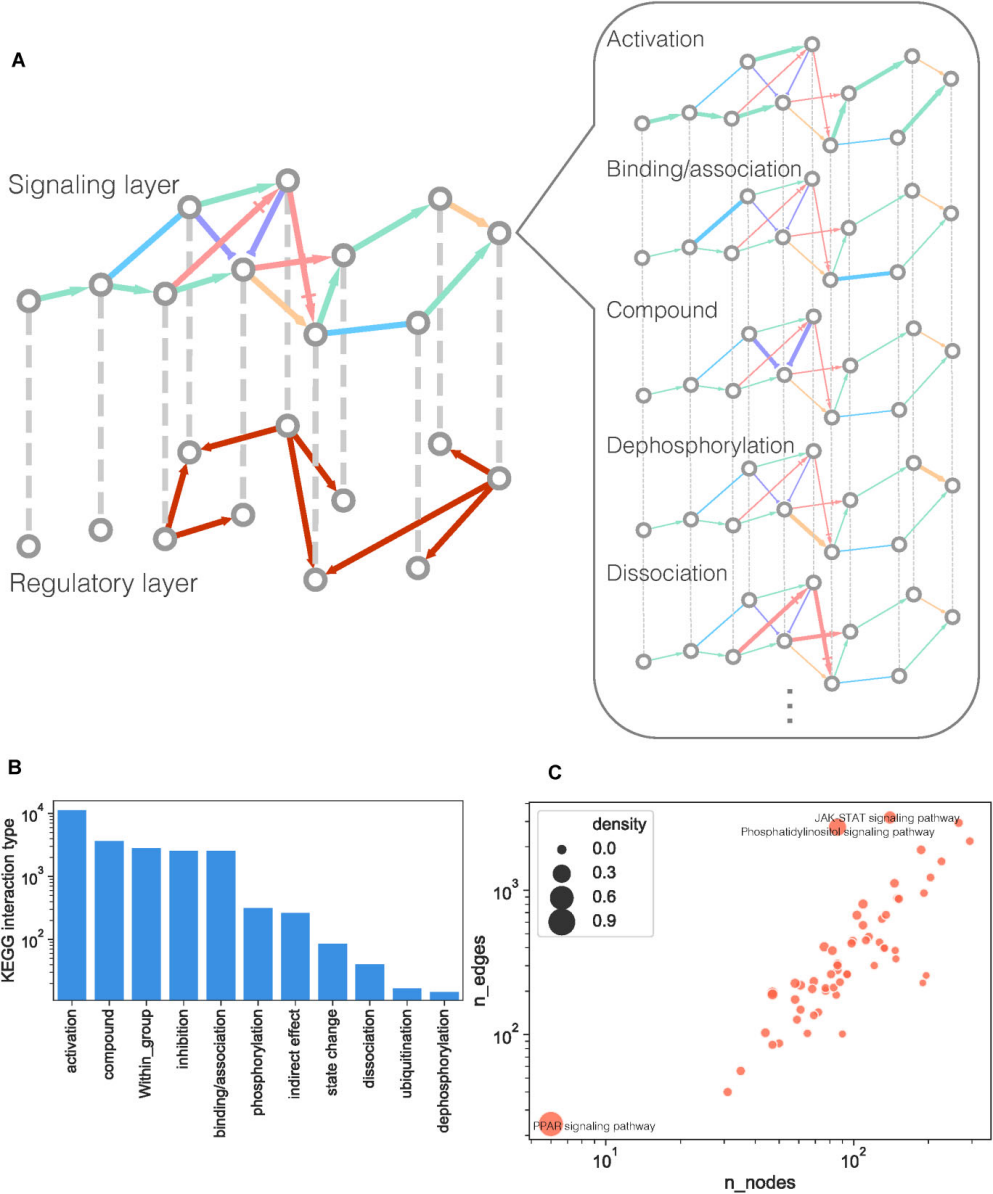
(**A**) Schematic showing the multilayer network consisting of a regulatory layer and a signaling layer, which in turn comprises individual layers for each type of signaling interaction in the KEGG signaling network, including a ‘context-free’ protein–protein interaction (PPI) network that acts as a scaffolding for signaling interactions. Due to multiple signaling pathways being superimposed, nodes can be connected by multiple parallel edges in the signaling layer. (**B**) The number of unique edges belonging to each signaling interaction type. (**C**) The number of nodes and edges for each KEGG signaling pathway. Circle sizes correspond to network density, defined by the ratio of the number of existing edges to the number of possible edges given the network size. Pathways with high network density (>0.1) are indicated.

To jointly analyze signal transduction and transcriptional regulatory interactions, we built a multilayer network that consists of a signaling layer and a regulatory layer (Figure 1A). We first constructed a network of known signaling events in human using signaling pathways curated from the KEGG database (33) (Materials and methods). The resulting KEGG signaling network, comprised of 60 signaling pathways (Supplementary Table S1), was a directed multigraph (i.e. a network that can have multiple directed edges between the same pair of nodes) with 2363 genes and 40 966 edges encompassing 11 interaction types (Figure 1B). Despite being diverse in size, with anywhere from 6 to 294 nodes, most signaling pathways had similar network densities (<0.1) (Figure 1C and Supplementary Table S1). The edges in the KEGG signaling network had high edge multiplicity (i.e. multiple edges between the same pair of nodes) with 18% of edges being present in multiple pathways, typically four or fewer (Supplementary Figure S1A). Furthermore, a sizeable portion (17%) of these edges (635 edges in total, corresponding to 3% of all signaling edges) represented different types of interactions in different pathways (Supplementary Figure S1B,C). As an example, the edge from NF1 to KRAS is inhibitory in the MAPK signaling pathway, whereas the same edge represents activation in the Ras signaling pathway.

While this directed network of annotated signaling pathways is necessary to provide biological context, it is, by itself, generally not sufficient for discovering new mediating interactions between signaling pathways (19). For this purpose, larger and unannotated (context-free) undirected protein–protein interaction (PPI) networks have often been used as a scaffolding, or ‘skeleton’ network, to underpin efforts to model an organism’s signaling circuitry (1,2,34). We supplemented the KEGG signaling network with a literature-curated, large-scale PPI network (24), whose combination with the KEGG network formed the signaling layer (Materials and methods). The addition of the PPI edges expanded the scope of the signaling network to 16 080 nodes and 239 048 edges. For the transcriptional regulatory layer, we used a previously published large-scale human gene regulatory network (GRN) (25). To ensure the robustness of our approach for networks with different densities, we used GRNs generated using three different *P*-value thresholds, which resulted in GRN densities spanning three orders of magnitude (Supplementary Table S2) (Materials and methods).

### Signaling-regulatory interface is located in the intermediary network region between signaling pathways.

**Figure 2.**
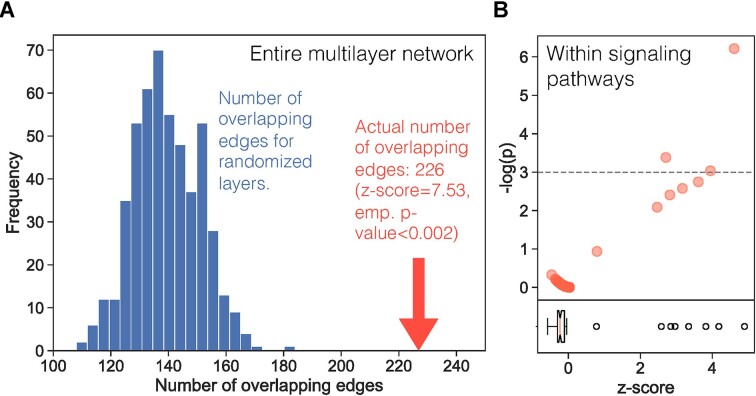
(**A**) The number of overlapping signaling and regulatory edges in the multilayer network with a GRN layer determined using a *P*-value threshold of *P*< 10^−6^. The blue bars show the distribution of overlapping edges for the randomized networks and the red arrow indicates the overlap for the actual multilayer network. (**B**) The -log(empirical *P*) values for overlap within each signaling pathway in the multilayer network with the GRN layer (*P*-value threshold of *P*< 10^−6^). Each dot represents a KEGG pathway. The boxplot indicates the distribution of z-scores for overlap within each pathway.

Overlapping signaling and regulatory interactions signify potential points of interface between the signaling and regulatory networks that might contribute to crosstalk (18). To quantify the degree of this interfacing, we measured the overlap between signaling and regulatory edges across the entire multilayer network. We compared the actual number of overlapping edges with the overlap observed between the two layers in randomized networks (Materials and methods). There were 226 edges common to both the signaling and the regulatory layer, which was significantly higher than random expectation (z-score = 7.53, empirical *P*-value < 0.002) (Figure 2A). This observation was independent of the density of the regulatory layer (Supplementary Figure S2A,C). In contrast with this overall edge overlap, the signaling and regulatory edge overlap within KEGG signaling pathways was not significant for the majority of pathways (median z-score = −0.23) (Figure 2B). Even with two denser GRNs in which a high layer overlap is expected, only about half of the pathways had significant overlap, with a median z-score of 1.13 and 2.98 (Supplementary Figure S2B,D). In total, a high proportion (83–93%) of signaling-regulatory interaction overlap (Supplementary Figure S3) was located outside of the signaling pathways. Together, these results imply that the significant signaling-regulatory interaction overlap is primarily located in the intermediary network region between the KEGG signaling pathways. Since signaling crosstalk is frequently mediated by the interactions between signaling pathways, these overlapping signaling-regulatory interactions that lie between pathways warrant closer investigation and might shed light on crosstalk mechanisms.

### Multilink statistics reveal mechanisms between signaling and regulatory interactions

**Figure 3.**
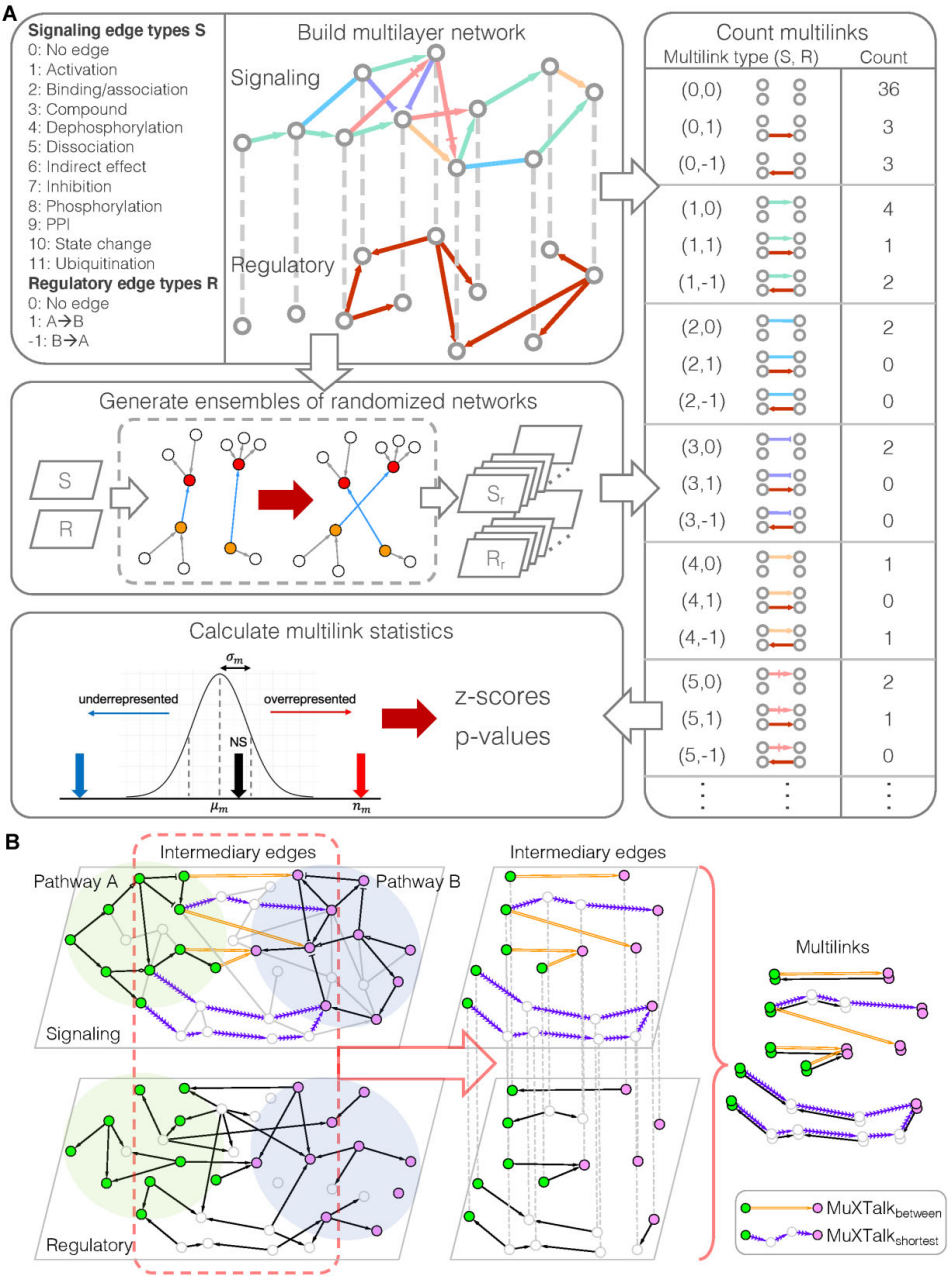
(**A**) Overview of the MuXTalk framework: The multilayer network comprises, in total, 36 multilink types $( {S,\ R} )$ where $S \in [ {0,\ 11} ]$ and $R \in \{ { - 1,\ 0,\ 1} \}$ are integers representing signaling and regulatory edge types. MuXTalk counts each multilink type for both the actual multilayer network and the randomized versions of the multilayer network. The actual multilink counts are then compared to the distribution of randomized counts, which results in multilink statistics (z-scores and *P*-values). ${S}_r$ and ${R}_r$ denote randomized instances of the signaling and regulatory layer, respectively. Note that, while we show in this schematic the counts for both instances of indistinguishable multilink types (e.g. $( {0,\ 1} )$ and $( {0,\ - 1} )$), MuXTalk avoids double-counting these types of multilinks (Methods). (**B**) MuXTalk identifies crosstalk based on the multilinks of the intermediary edges connecting Pathway A and Pathway B. In particular, MuXTalk_between_ and MuXTalk_shortest_ use direct edges and shortest paths between pairs of pathways, respectively, as intermediary edges to detect crosstalk.

To integrate signaling and regulatory interactions, we rely on high-dimensional edges called *multilinks* (22,23), which enable us to simultaneously keep track of multiple types of concurrent and context-dependent interactions present in a multilayer network. Concretely, multilinks enumerate every possible combination of interaction types across all layers of the multilayer network as a unique type of edge, each of which exemplifies a distinct signaling/regulatory mechanism (Figure 3A). We hypothesized that the relative abundance of multilink types can be used to assess the contribution of each mechanism to a given signaling pathway and to subsequently infer crosstalk between signaling pathways. We developed a statistical framework, named MuXTalk, that (i) determines whether each multilink type is over- or under-represented in the multilayer network and (ii) identifies potentially crosstalking pairs of pathways using a certain class of multilinks as a proxy of crosstalk. Briefly, MuXTalk performs the following steps (Figure 3): (i) It counts the number of occurrences of each multilink type in the multilayer network; (ii) It compares these numbers to those obtained from a large ensemble of degree-preserved randomized networks to calculate statistics (z-scores and empirical *P*-values) associated with each multilink type; (iii) It prioritizes pairs of pathways as the most likely to crosstalk using the multilink statistics of the edges between signaling pathways (detailed in Materials and methods and the following section).

**Figure 4.**
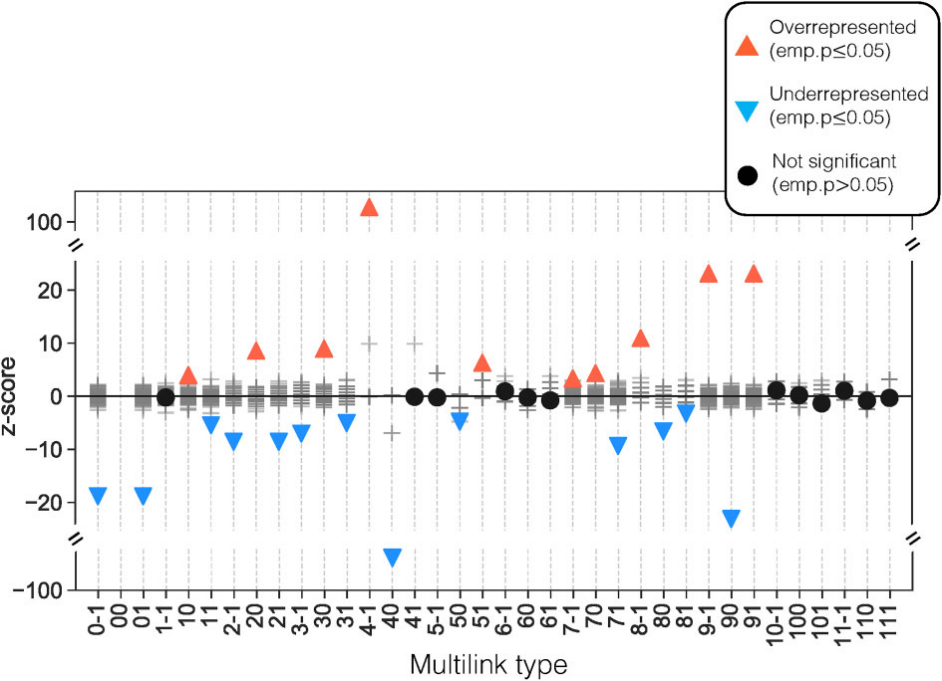
Multilink profiles of all edges in the multilayer network with a GRN layer based on a *P*-value threshold of *P*< 10^−4^. Multilink types are represented by numerical indicators where the first one or two digits represent the signaling edge type and the last digit represents the regulatory interaction direction. Red triangles pointing up and blue triangles pointing down indicate statistically over-represented (*z* > 0; emp. *P*$ \le$0.05) and under-represented (*z* < 0; emp. *P*$ \le$0.05) multilink types, respectively. Black circles denote statistically insignificant multilink types (emp. *P*$ >$0.05).

For a more detailed picture of signaling and regulatory overlap across all KEGG signaling pathways, we first used MuXTalk to calculate the multilink statistics of all edges in the multilayer network (Figure 4). Overall, multilinks of type $( {0,\ 1} )$ and $( {0,\ - 1} )$, which represent the absence of an edge in the signaling layer and the presence of an edge in the regulatory layer (Figure 3A), were significantly under-represented across all signaling pathways, supporting our finding of significant overlap between the two layers in the previous section. When broken down into individual signaling events, dephosphorylation, dissociation, inhibition, phosphorylation and protein–protein interactions were often accompanied by regulatory interactions. Dephosphorylation, inhibition and phosphorylation events were in opposite direction with regulatory events (i.e. $( {4,\ - 1} )$, $( {7,\ - 1} )$ and $( {8,\ - 1} )$ multilink types were significantly over-represented), whereas dissociation events were in the same direction as regulatory events (i.e. $( {5,\ 1} )$ was significantly over-represented). In contrast, activation, binding/association, and compound interactions tended to occur without concurrent regulatory interactions (i.e. multilink types $( {1,\ 0} )$, $( {2,\ 0} )$ and $( {3,\ 0} )$ were over-represented. These results are concordant with signaling-regulatory mechanisms documented in the literature: Phosphorylation and dephosphorylation can directly regulate transcription factor function (35,36); inhibition of certain pathways has been shown to antagonize transcription factor function (37); the dynamics of dissociation of TFs from their targets have been shown to determine their function (38); activators tend to be co-activator TFs and are therefore generally not targets of TFs.

Notably, the over- and under-represented multilink types were consistent across GRNs through the entire range of densities tested, with the exception of some missing multilinks due to the limitations of calculating multilink statistics in sparser GRNs (Supplementary Figure S4). Further, the significantly over- and under-represented multilinks within individual signaling pathways were highly pathway-specific, showing high variability in terms of which mechanisms were predominantly featured in each pathway, including more ‘elusive’ mechanisms such as ubiquitination and state change (Supplementary Figures S5–7). Together, these results suggest that multilink statistics can be a useful tool in extracting signaling-regulatory mechanisms within and between signaling pathways, offering clues as to which mechanisms may be involved in signaling crosstalk.

### MuXTalk outperforms other methods in identifying crosstalk

After exploring the usefulness of multilinks in elucidating signaling-regulatory mechanisms, we next tested our hypothesis that multilink statistics of edges in the intermediary region between signaling pathways can be harnessed to identify their crosstalk. In particular, we used the over-representation of multilink types that include regulatory edges (i.e. multilink types $( {S,\ 1} )$ and $( {S,\ - 1} )$, see Figure 3A and Materials and methods) among edges connecting a given pair of pathways as a proxy of their crosstalk. With this definition of crosstalk, we can capture, at once, multiple mechanisms through which crosstalk can occur, including directed signaling interactions and undirected protein-protein interactions, as well as regulatory mechanisms such as transcription factors of a pathway targeting members of another pathway (39,40) and ensuing feedback loops (41–43). Since crosstalk can occur when signaling pathways connect either directly or indirectly (3), we implemented two complementary approaches within MuXTalk, namely, MuXTalk_between_ and MuXTalk_shortest_ (Figure 3B), which consider the direct edges and long-range interactions (i.e. shortest paths) between pathways, respectively (Materials and methods).

**Figure 5.**
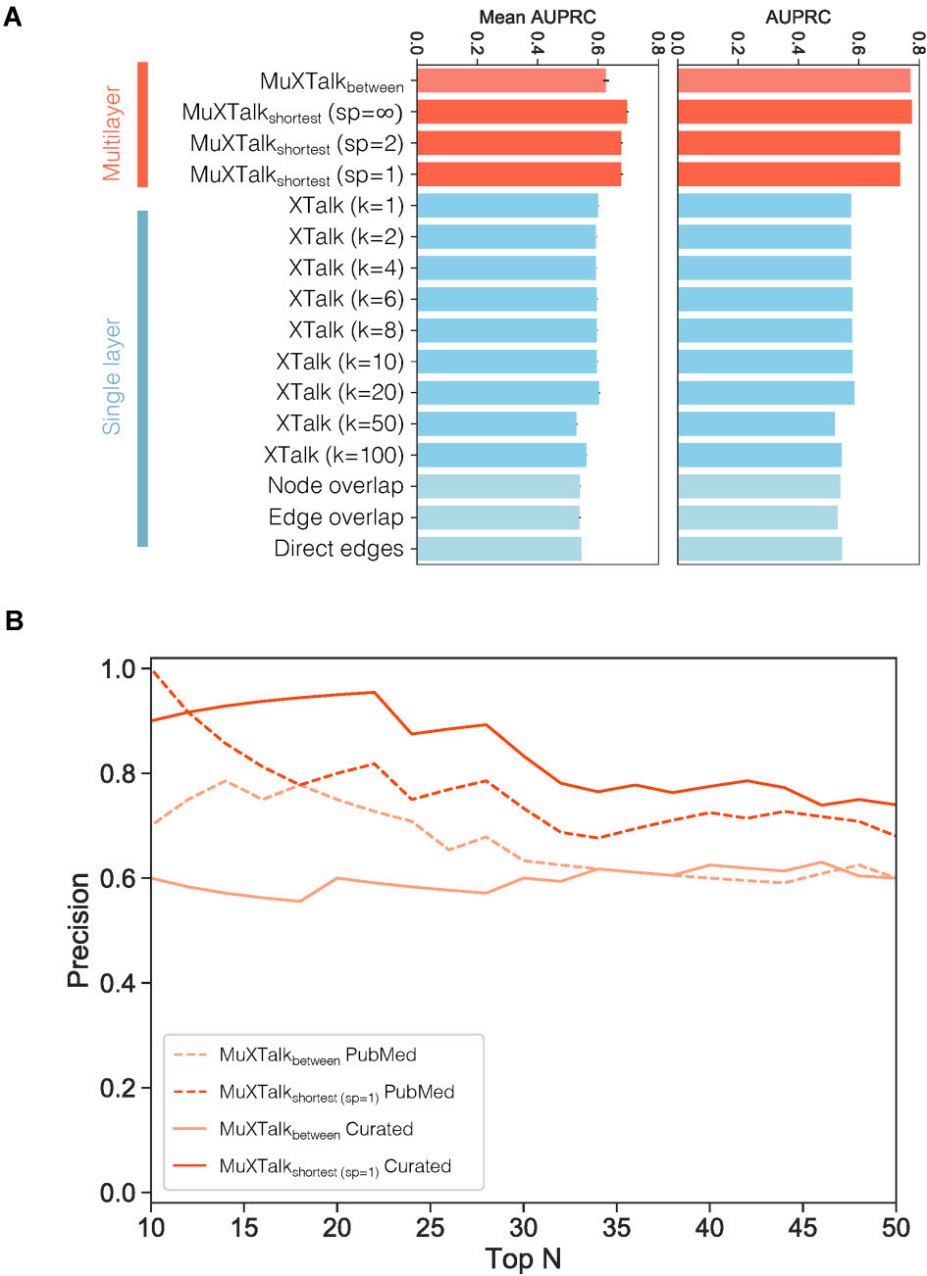
(**A**) Area under the precision-recall curves (AUPRC) for MuXTalk (red) and four other methods (blue) for the stochastic (left) and deterministic (right) versions of the benchmark. Error bars indicate the standard deviation. MuXTalk was run on the multilayer network using a GRN layer based on a *P*-value threshold of *P*< 10^−6^ (**B**) Precision-rank plot for the top 50 inferences in the discovery set of pathways. Dashed and solid lines are for PubMed query only and PubMed query-guided manual curation, respectively.

To assess the validity of the MuXTalk inferences and compare the model performance to those of other methods, we designed two benchmarks, one deterministic and one stochastic, based on crosstalking pathway pairs from the literature-curated crosstalk database XTalkDB (27) (Materials and methods). Since XTalkDB only includes 25 of the 60 KEGG signaling pathways considered here, we used these 25 pathways as our ‘benchmark set’ and the remaining pathways as our ‘discovery set’ (Supplementary Table S1). As exemplary methods to compare MuXTalk with, we chose four other network-based approaches that rely on node and edge overlap (8,9), direct edges (13,28) and shortest paths (16) between pathways. Both versions of MuXTalk surpassed all other methods in terms of the area under both the receiver operating characteristic (ROC) curve and the precision-recall (PR) curve, in both the deterministic and the stochastic version of the benchmark (Figure 5A and Supplementary Figure S8). The differences in the above performance metrics were all statistically significantly higher when using MuXTalk as compared to the other methods (Supplementary Table S4). These results were recapitulated when we used denser GRNs (Supplementary Figure S9), suggesting the robustness of MuXTalk against changes in network density. Despite a slight drop in the performance with the densest GRN, we see its sustained advantage over alternative methods (Supplementary Figure S9). To measure the effect of using different data sources on overall model performance, we used two additional network resources, namely, STRING (44) and GRNdb (45) for the PPI component of the signaling layer and the GRN layer, respectively (Materials and methods). Incorporating these networks in MuXTalk resulted in performance metrics that were generally comparable with, if slightly lower than, those of our original networks and higher than those of the other methods used in the benchmark (Supplementary Figure S10). The relatively lower performance of the high-confidence STRING network (confidence score > 900) can be attributed to its smaller size (∼9200 nodes), which has an overall impact on the deterministic benchmark values due to an increased number of pathway elements not present in the multilayer network, precluding crosstalk assessment by MuXTalk. Together, these sensitivity analyses suggest the robustness of MuXTalk to different network sources while retaining its advantage over other available methods. Finally, a leave-one-layer-out cross-validation (Materials and methods) revealed that the overall model performance as measured by the area under the ROC and PR curves was generally highly robust to the exclusion of a single signaling layer (Supplementary Table S5). This robustness was more pronounced for the sparser GRNs since the vast majority of inferences in this case were driven by multilinks with either a PPI edge or no edge on the signaling layer, and for a small minority by an activation edge. With denser GRNs, a more discernable, yet still subtle, difference emerged between the AUC values, where the exclusion of the binding/association layer resulted in slightly lower AUC values compared to the other layers.

### Discovering new putative crosstalk connections with MuXTalk

Encouraged by the benchmark performance of MuXTalk compared to other network-based methods, we next used MuXTalk on the ‘discovery’ set of pathway pairs to identify potential signaling crosstalk between pathways not included in the benchmark set. Unlike our benchmark set of pathway pairs, the discovery set does not have crosstalk data that we can use as the ground truth. Therefore, we devised an approach that combines automated PubMed queries with manual curation to capture potential crosstalk (Materials and methods, Supplementary Files S1–2). Based on the 50 top-ranked pathway pairs identified by MuXTalk_shortest_ and MuXTalk_between_, these methods showed a mean precision of 79% and 66%, respectively, for the automated PubMed query only, and a mean precision of 84% and 60%, respectively, for PubMed query-guided manual curation (Figure 5B). The automated PubMed query results for the 400 top-ranked pathways is shown in Supplementary Figure S11. Together, these results provide support for the novel crosstalk predictions made by MuXTalk.

### MuXTalk sheds light on crosstalk mechanisms; identifies potentially novel mediators of crosstalk

To demonstrate MuXTalk's potential utility for discovering novel crosstalk events and providing mechanistic insights to each, we focused on the highest ranked crosstalking pairs identified by MuXTalk. Both MuXTalk_between_ and MuXTalk_shortest (sp=1)_ identified the crosstalk between the neurotrophin signaling pathway and the TGF-β signaling pathway among the top-ranked crosstalking pathway pairs previously not documented in the benchmark data (Supplementary Tables S7 and 8).

**Figure 6.**
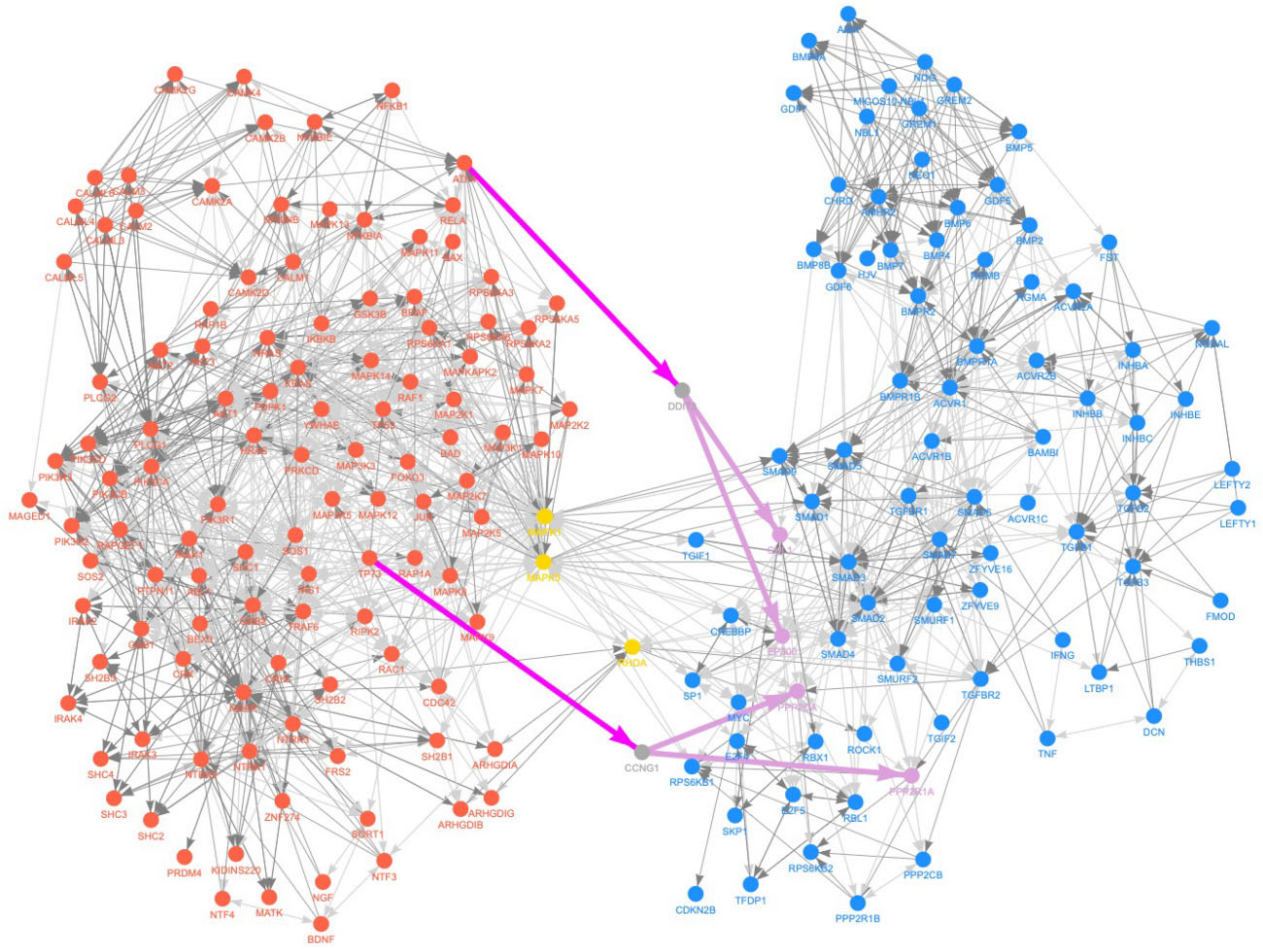
Example output from the MuXTalk web app that shows the crosstalk from the neurotrophin signaling pathway (red) to the TGF-β signaling pathway (blue), inferred by MuXTalk_shortest_ (with sp = 1). Orange nodes indicate the genes common to both pathways. Purple edges indicate significant multilinks of type $( {9,\ - 1} )$ (PPI edge and a GRN edge from the neurotrophin signaling pathway to the TGF-β signaling pathway) that were identified by MuXTalk to mediate the crosstalk.

While the crosstalk from the TGF-β signaling pathway to the neurotrophin signaling pathway, as captured by MuXTalk_between_, has been previously established due to the transcriptional regulatory role of TGFβ signaling on neurotrophins (46–48) the crosstalk in the opposite direction has been relatively underappreciated until recently (49) wherein it was speculated that neurotrophins and TGF-βs act in concert to activate a mutual protective signaling network. Supporting this notion, MuXTalk_shortest_ discovered two statistically significant multilinks of type $( {9,\ 1} )$ (meaning a PPI and a regulatory interaction, see Figure 3A) pointing from the neurotrophin signaling pathway to the TGF-β signaling pathway (Figure 6), connecting Activating Transcription Factor 4 *(ATF4)* with DNA Damage Inducible Transcript 3 *(DDIT3)* and Tumor Protein P73 *(TP73)* with Cyclin G1 *(CCNG1)*. *DDIT3* and *CCGN1* here serve as the intermediary molecules that interact with E1A Binding Protein P300 *(EP300)* and Cullin 1 *(CUL1)* and Protein Phosphatase 2 Catalytic Subunit Alpha *(PPP2CA)* and Protein Phosphatase 2 Scaffold Subunit Aalpha *(PPP2R1A)*, respectively, in the TGF-β signaling pathway.

The impact of *DDIT3* (also known as the growth arrest and DNA damage-inducible protein 153 *(GADD 153)* and encodes the C/EBP homologous protein (*CHOP*)) in diverse human diseases including neurodegenerative disorders has been established, whereas the emerging role of *DDIT3* in fibrosis has only recently been reported (50). In particular, the crucial role of *DDIT3*/*CHOP* expression in the negative regulation of two neurotrophic cytokines, leptin and insulin-like growth factor-1, has been reported (51), whereas other mechanistic studies showed that *CHOP* regulates the production of pro-inflammatory (M2) macrophages and subsequent TGF-β signaling involved in idiopathic pulmonary fibrosis (IPF) (52). In turn, the inhibition of the two interactions of *DDIT3* identified by MuXTalk, *CUL1* and *EP300*, has been shown to reduce fibroblast proliferation in chronic obstructive pulmonary disease (COPD) and IPF (53,54).

TP73 is a member of the p53 family of transcription factors that targets neurotrophin receptor (p75NTR), promoting terminal neuronal differentiation (55,56). Neurotrophin signaling mediated through p75NTR has been shown to activate the nuclear factor-kB (NF-kB) and Jun kinase signaling pathways (including JNK pathway) (57,58). While the NF-kB and JNK pathways play an important role in neural development in many aspects, it is also known that they regulate the expression and activation of cyclins, including Cyclin G1 (CCNG1) (59–61). More specifically, the NF-kB signaling pathway was shown to regulate Cyclin D1 (CCND1) expression, whereas the JNK signaling pathway was shown to regulate the expression of both CCND1 and CCNG1 (59–61). CCNG1 is a non-canonical cyclin that was initially discovered as a novel member of the cyclin family (62). Although the exact mechanism is not yet clear, CCNG1 is known to play a crucial role in cell proliferation and cell growth (63–65). Indeed, CCNG1 was shown to directly interact with Protein Phosphatase 2A (PP2A), which is a holoenzyme complex that consists of the PPP2CA and PPP2R1A subunits, and recruits PP2A to MDM2 (62,66–69). The PP2A mediated de-phosphorylation and activation of MDM2, results in the destabilization of P53, which is crucial in cell proliferation, signal transduction and apoptosis (62,68,69). While PP2A may influence TGF-β signaling through P53 indirectly, PP2A was also shown to directly regulate TGF-β signaling by de-phosphorylating the TGF-β receptor (70,71).

To provide orthogonal support for the directional crosstalk between neurotrophin and TGF-β signaling pathways, we used chromatin immunoprecipitation sequencing (ChIP-seq) data from ChIP-Atlas (72) to identify the potential transcriptional regulators of TGF-β signaling pathway by measuring the enrichment of ChIP-seq peaks corresponding to transcription factor (TF) regulatory elements (Materials and methods). Three TFs in the neurotrophin signaling pathway (JUN, TP53, RELA) had a significant (two-sided Fisher’s exact *P*-value < 0.05, fold-enrichment > 1.4) enrichment of TF-regulatory peaks in the TGF-β signaling pathway (Supplementary Figure S12). In addition, seven other TFs in the neurotrophin signaling pathway, including ATF4 and TP73, had a fold-enrichment > 1.4, even though these were below nominal significance. Together, these results further support our highlighted example through the statistical enrichment of ChIP-seq TF peaks.

## Discussion

Signaling pathways combine with and modulate each other in myriad ways in an intricate web of signaling and regulatory interactions, allowing cells to fine-tune their responses to their microenvironment. Canonical signaling pathways and their crosstalk have long been subjects of targeted studies that derive mechanistic insights in the context of a disease or developmental process of interest. Given the combinatorial space within which signaling pathways can interact, both at the physical protein interaction and transcriptional regulation level, untargeted computational methods can provide a holistic view of crosstalk by analyzing the global network of signaling interactions and offering putative crosstalk mechanisms in a context-specific way. In this study, we introduced a multilayer network-based statistical framework to integrate signaling and regulatory interactions, which allowed us to infer crosstalk events and identify their potential mediators.

Dedicated manual curation efforts have resulted in highly reliable crosstalk databases such as XTalkDB that continue to inform computational efforts (16), including ours. Still, by definition, such studies rely on existing knowledge: They use as their starting point keyword searches in PubMed and then filter these automated results based on expert curation. As such, they have the potential to miss the subtler ways in which signaling crosstalk can be described in the literature. Our approach, by contrast, takes the molecular bases of crosstalk as the starting point and identifies the potential mediators of crosstalk, potentially transcending the limitations of what is already published in the literature and uncovering novel mechanistic insights. Indeed, many of the studies supporting our use cases have been published after these crosstalk databases were established, suggesting the potential complementary role of ‘prospective’ computational models to ‘retrospective’ literature curation.

Statistical methods that can distinguish meaningful signals from the noise rampant in biomedical data (73,74) ought to be preferred where possible. In the context of biological networks, combating noise translates into crafting null network models that generate relevant controls (75–77). When we are interested in the edges that connect signaling pathways as proxies of crosstalk, a highly pertinent question to ask is whether the observed number of connecting edges is more or less than what would be expected by chance, given the connectivity structure of each network. The recently published version of SignaLink (78), which identifies signaling crosstalk and is similar to our work in that it is integrative and offers context-specificity, differs from our method in this important aspect: It is not a statistical framework; it does not perform any hypothesis testing but, rather, simply chronicles the number of connecting edges, and hence does not provide a sense of whether the number of edges between a pair of pathways is statistically meaningful within the underlying network of interactions. With MuXTalk, we generate interaction type-specific ensembles of randomized networks that act as null models and provide a standardized backdrop against which the number of edges can be compared, ensuring that the statistically over-represented multilinks are not simply byproducts of implicit and systematic data biases (79,80).

One of the limitations of our approach is that it fundamentally relies on knowledge repositories where genes and their products are organized into canonical pathways according to their functional annotations, which themselves might be incomplete (81). There are several such pathway databases with complementary strengths, although traditionally, unifying them has been deemed nontrivial due to varying standard formats and data models (82). As a result, the choice of which database to employ is often based on the specifics of each use case. We chose KEGG for our framework since it is widely used and recognized, has high granularity in terms of identifying distinct interaction types, and can be directly harmonized with XTalkDB for benchmarking. Although it is certain that database choice has at least some bearing on downstream analyses, there is emerging evidence that the disparities between pathway resources might be less consequential than previously estimated: Pathways across different databases were recently found to display much higher levels of agreement when database structural differences were corrected for (83). Finally, although topology-based approaches such as ours have been shown to have relatively less bias on KEGG when mechanistic relationships are investigated (84), we acknowledge the inherent limitations of KEGG and other pathway resources in that they are highly heterogeneous in terms of data sources, cell types, models, and annotation quality, and that the results presented in this work should be interpreted in this light. It is an ongoing challenge and an open question how to account for pathway database biases in methods like ours.

Another limitation is the infeasibility of performing an exhaustive literature curation for our discovery analysis. While automated PubMed queries yield a potentially credible set of publications supporting crosstalk, a rigorous validation cannot be carried out without inspecting the corresponding PubMed hits for crosstalk-relevant content. Given the dozens to hundreds of PubMed IDs returned for each query, we elected to do this labor-intensive manual curation for the top 50 queries for each MuXTalk method. Emerging generative large language models that are domain-specific to biomedicine, such as BioGPT (85), might facilitate the automation of such biomedical knowledge curation processes in the near future.

Computational constraints generally force a tradeoff between detailed yet small-scale models that simulate signaling dynamics under different perturbations and large-scale yet static models that describe signaling pathways in terms of their topological features. As an example of the latter class of approaches, our method has the inherent limitation of not accounting for the dynamics and differing timescales of signaling and regulatory events. What MuXTalk lacks in this aspect, it makes up for in terms of the size of the multilayer network, spanning multiple types of interactions between over 16 000 proteins and 60 pathways. The study of crosstalk, in particular, is more amenable to this kind of a large-scale network-based approach since it involves interactions among pathways in the overall signaling network. That being said, scalable dynamic models, both fine-grained (atomistic) and coarse-grained (Boolean), still are indispensable vehicles to understand signaling networks and their crosstalk mechanistically. Several advancements in this area have been made in the past decade: Atomistic motif-based models of crosstalk have been proposed (86) and constraint-based stoichiometric approaches akin to the ones used in metabolic networks have been developed for signaling networks (87). Even though these methods are typically applied on individual signaling pathways, the near future may see the fusing of such dynamical models with global approaches like ours.

MuXTalk supports, by design, the discovery of context-specific crosstalk due to its incorporation of GRNs. The MuXTalk framework allows the input of custom GRNs, and we encourage users to explore this direction with GRNs derived from a certain tissue, cell type or disease, or even with condition-specific GRNs inferred from single-cell expression data (88). Aside from the custom GRN functionality, context-specificity can be further folded into the MuXTalk framework by using tissue- or disease-specific PPIs. In its simplest form, data from transcriptomics or proteomics experiments can be used (89) to construct context-specific PPIs whereby global PPIs are refined by only keeping the edges between genes or proteins expressed in a given tissue or disease, as done recently in, e.g. (90,91). However, dedicated benchmarks would be necessary to establish the context-specific use of our approach. It is currently challenging to develop benchmarking strategies for cell type- or disease-specific crosstalk due to the lack of ground truth repositories for context-specific crosstalk. This will undoubtedly become a promising future direction as advances in text mining and natural language processing for biomedical literature (92) gradually allow for context-specific knowledge bases for crosstalk.

## Supplementary Material

gkad1035_Supplemental_Files

## Data Availability

The source code and instructions are available in Zenodo at https://doi.org/10.5281/zenodo.10018775. The multilayer network data underlying this article are available in the Network Data Exchange (NDEx) portal (https://www.ndexbio.org/), at https://doi.org/10.18119/N9JS5K.
