# Supplementary material for: Detecting and dissecting signaling crosstalk via the multilayer network integration of signaling and regulatory interactions
Source: Nucleic Acids Res. 2023 Nov 11;52(1):e5. doi: 10.1093/nar/gkad1035 (PMC10783515; doi:10.1093/nar/gkad1035)
Supplement: gkad1035_Supplemental_Files [file gkad1035_supplemental_files.zip › MuXTalk_Supplementary_Figures_R2.pdf]

Supplementary Figures and Tables

Detecting and dissecting signaling crosstalk via the multilayer network integration of signaling and regulatory interactions

Leonardo Martini[1,2†], Seung Han Baek[3†], Ian Lo[4], Benjamin A. Raby[3], Edwin K. Silverman[1], Scott T. Weiss[1], Kimberly R. Glass[1,4^], Arda Halu[1^*]

[1]Channing Division of Network Medicine, Department of Medicine, Brigham and Women's Hospital, Harvard Medical School, Boston, USA
[2]Department of Computer, Control, and Management Engineering, Sapienza University of Rome, Rome, Italy
[3]Division of Pulmonary Medicine, Boston Children's Hospital, Harvard Medical School, Boston, USA
[4]Department of Biostatistics, Harvard T.H. Chan School of Public Health, Boston, USA

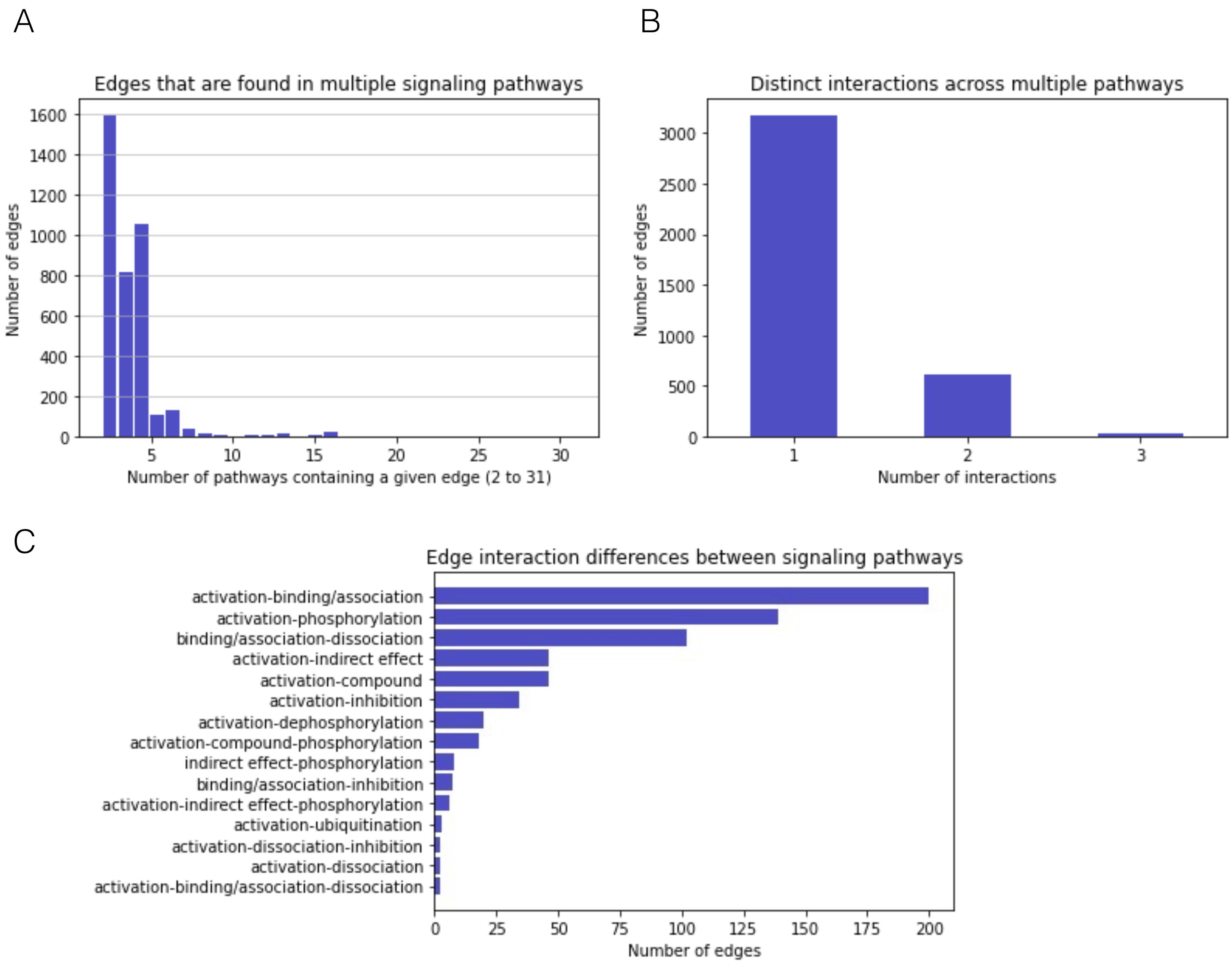

Supplementary Figure 1: (A) Distribution of the number of pathways of which each edge is a member. **(B)** The disctribution of the number of distinct interaction types in cases where the edge belongs to multiple pathways. **(C)** The breakdown of distinct interaction types in cases where the edge represents multiple distinct types of interactions across multiple pathways.

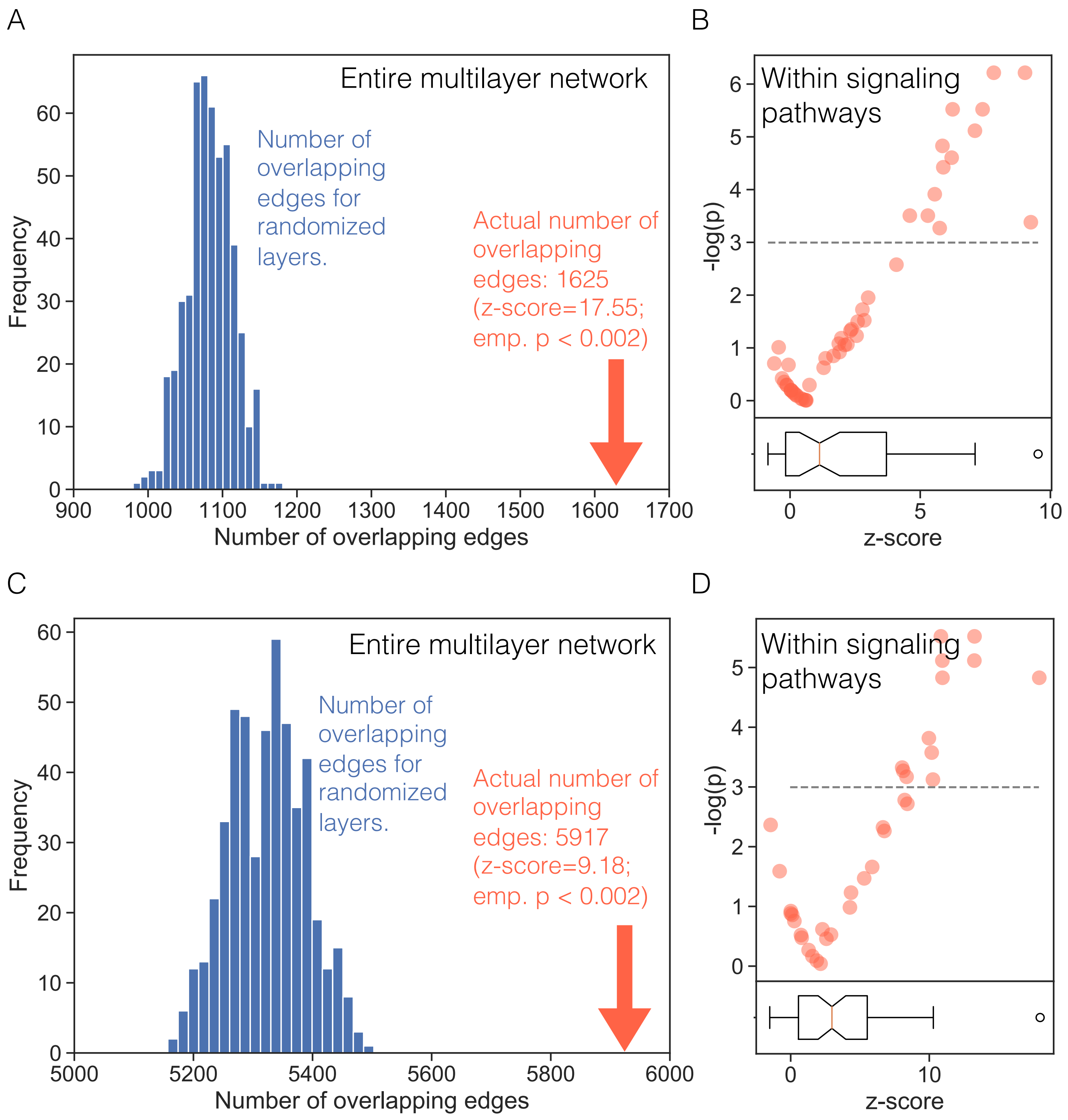

Supplementary Figure 2: (A, C) The number of overlapping signaling and regulatory edges in the multilayer network with a GRN layer of p-value threshold (p<10-5) and (p<10-4), respectively. The blue bars show the distribution of overlapping edges for the randomized networks and the red arrow indicates the overlap for the actual multilayer network. **(B, D)** The -log(empirical p) values for overlap within each signaling pathway in the multilayer network with a GRN layer of p-value threshold (p<10-5) and (p<10-4), respectively. Each dot represents a KEGG pathway. The boxplot indicates the distribution of z-scores for overlap within each pathway.

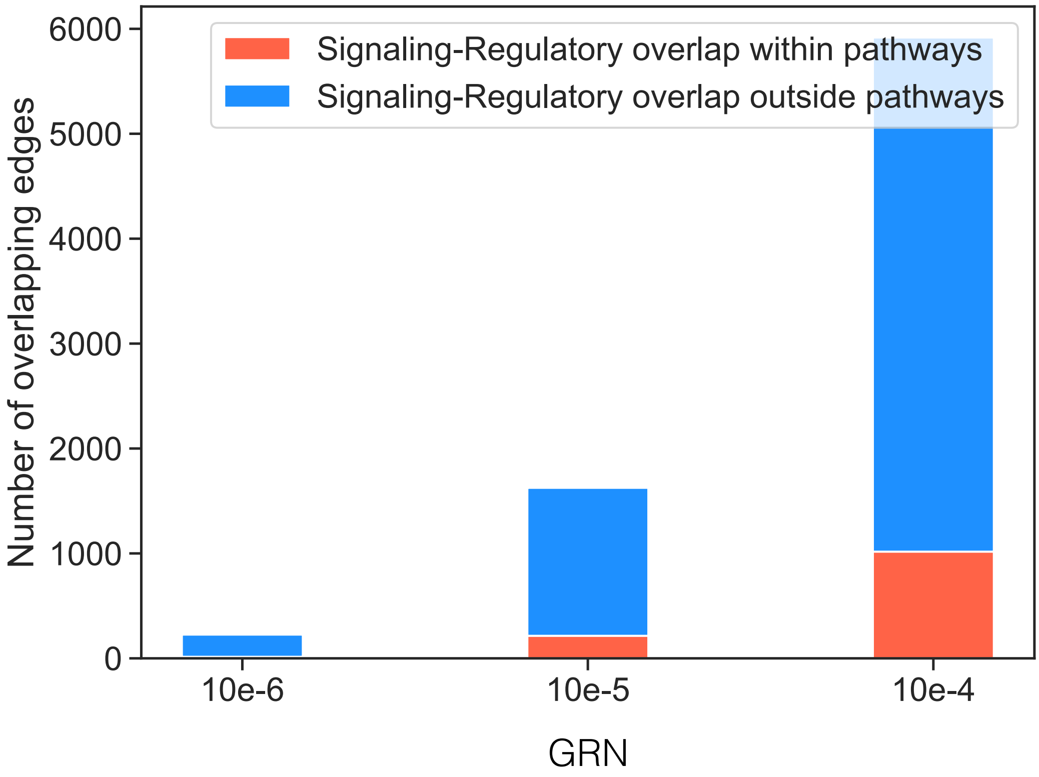

Supplementary Figure 3: The number of overlapping edges between the signaling and the regulatory layer for GRNs with different p-value thresholds. Blue bars indicate the proportion of overlap outside, or between, KEGG signaling pathways and the red bars indicate the proportion within KEGG signaling pathways.

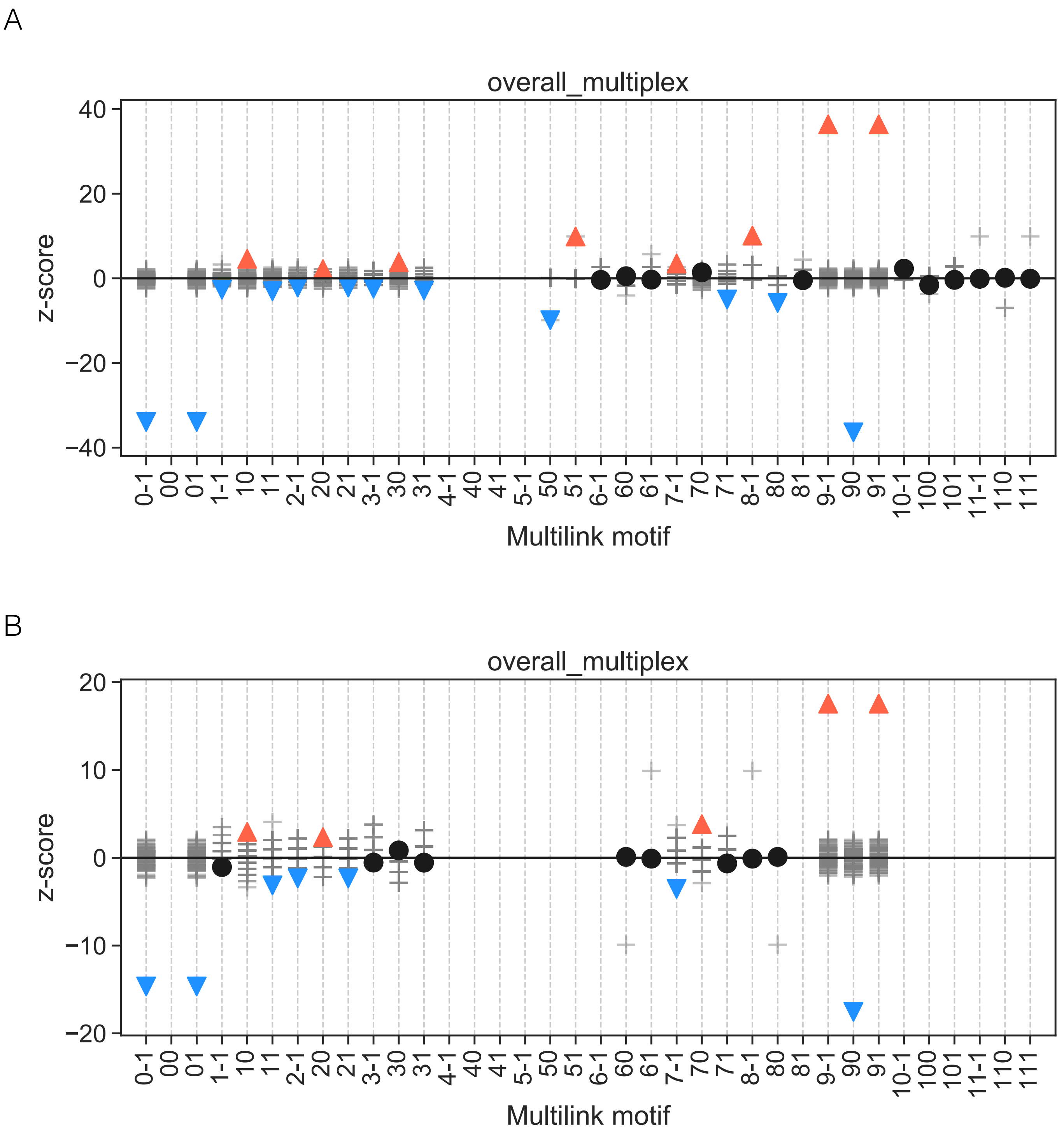

Supplementary Figure 4: Multilink profiles of all edges in the multilayer network with a GRN layer of p-value threshold (p<10-5) **(A)** and (p<10-6) **(B)**. Multilink types are represented by numerical indicators where the first one or two digits represent the signaling edge type and the last digit represents the regulatory interaction direction. Red triangles pointing up and blue triangles pointing down indicate statistically over-represented (z>0; emp. p≤0.05) and under-represented (z<0; emp. p≤0.05) multilink types, respectively. Black circles denote statistically insignificant multilink types (emp. p>0.05).

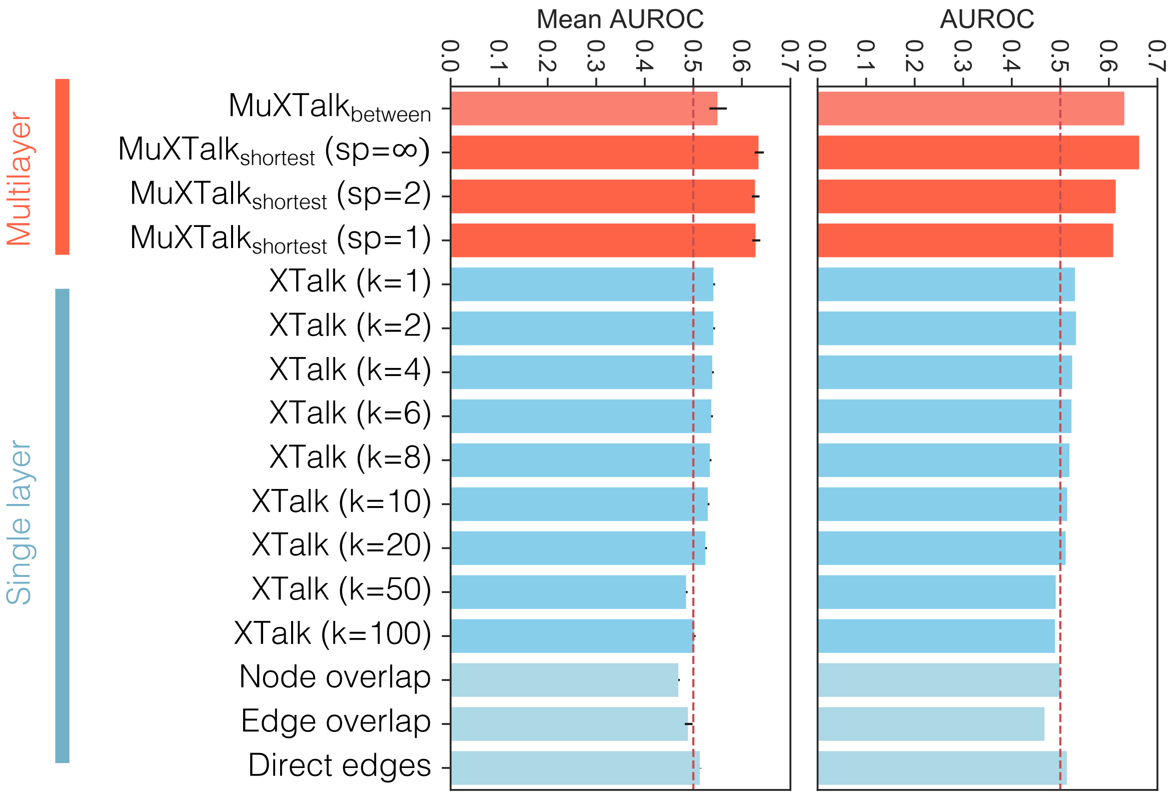

Supplementary Figure 8: Area under the receiver operating characteristic (AUROC) curves for MuXTalk (red) and four other methods (blue) for the stochastic (left) and deterministic (right) versions of the benchmark. Error bars indicate the standard deviation. MuXTalk was run on the multilayer network with a GRN layer of p-value threshold (p<10-6)

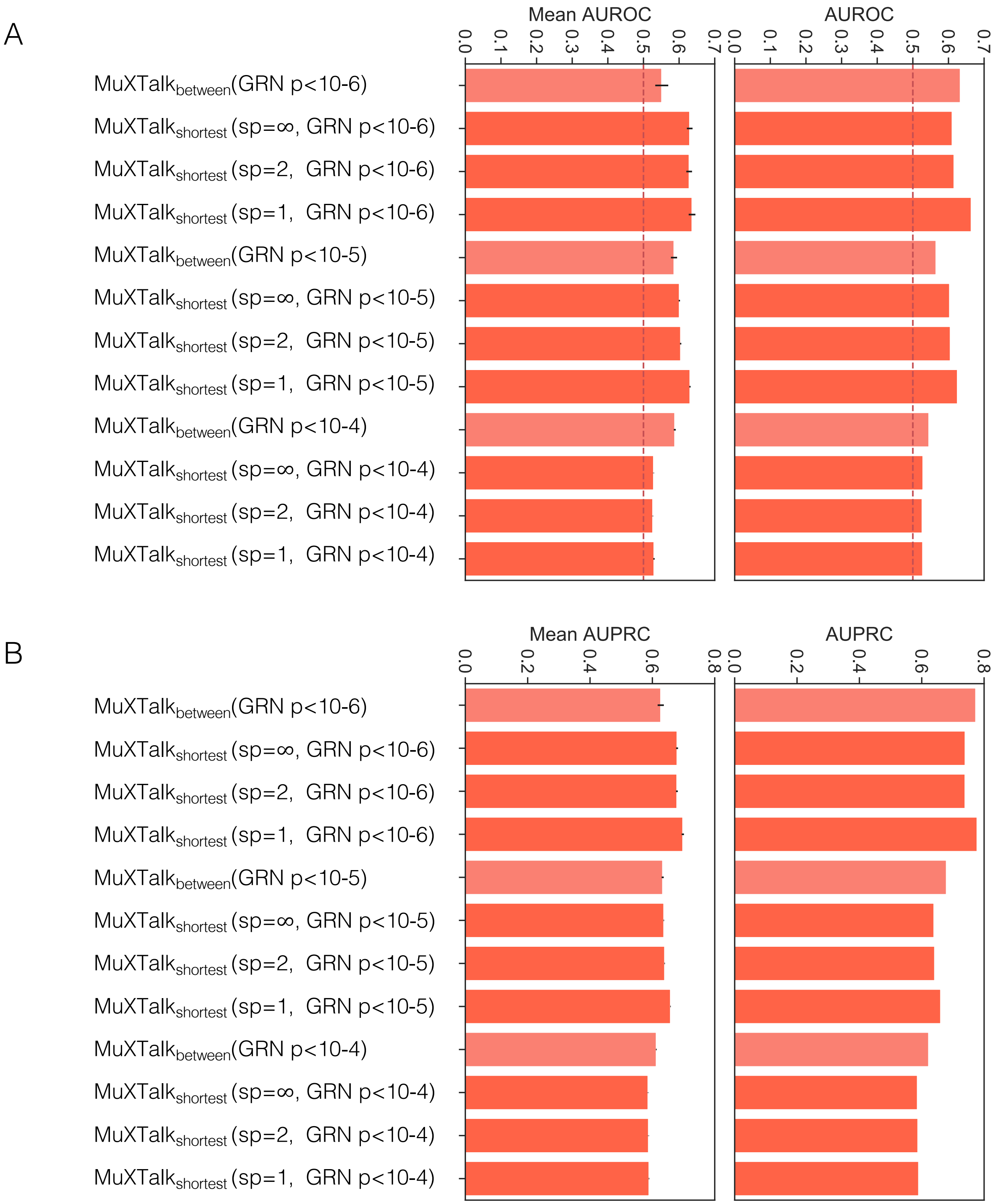

Supplementary Figure 9: Area under the receiver operating characteristic (AUROC) **(A)** and precision-recall curves (AUPRC) **(B)** for MuXTalk for the stochastic (left) and deterministic (right) versions of the benchmark, for multilayer networks with GRN layers of p-value threshold (p<10-6, p<10-5 and p<10-4). Error bars indicate the standard deviation.

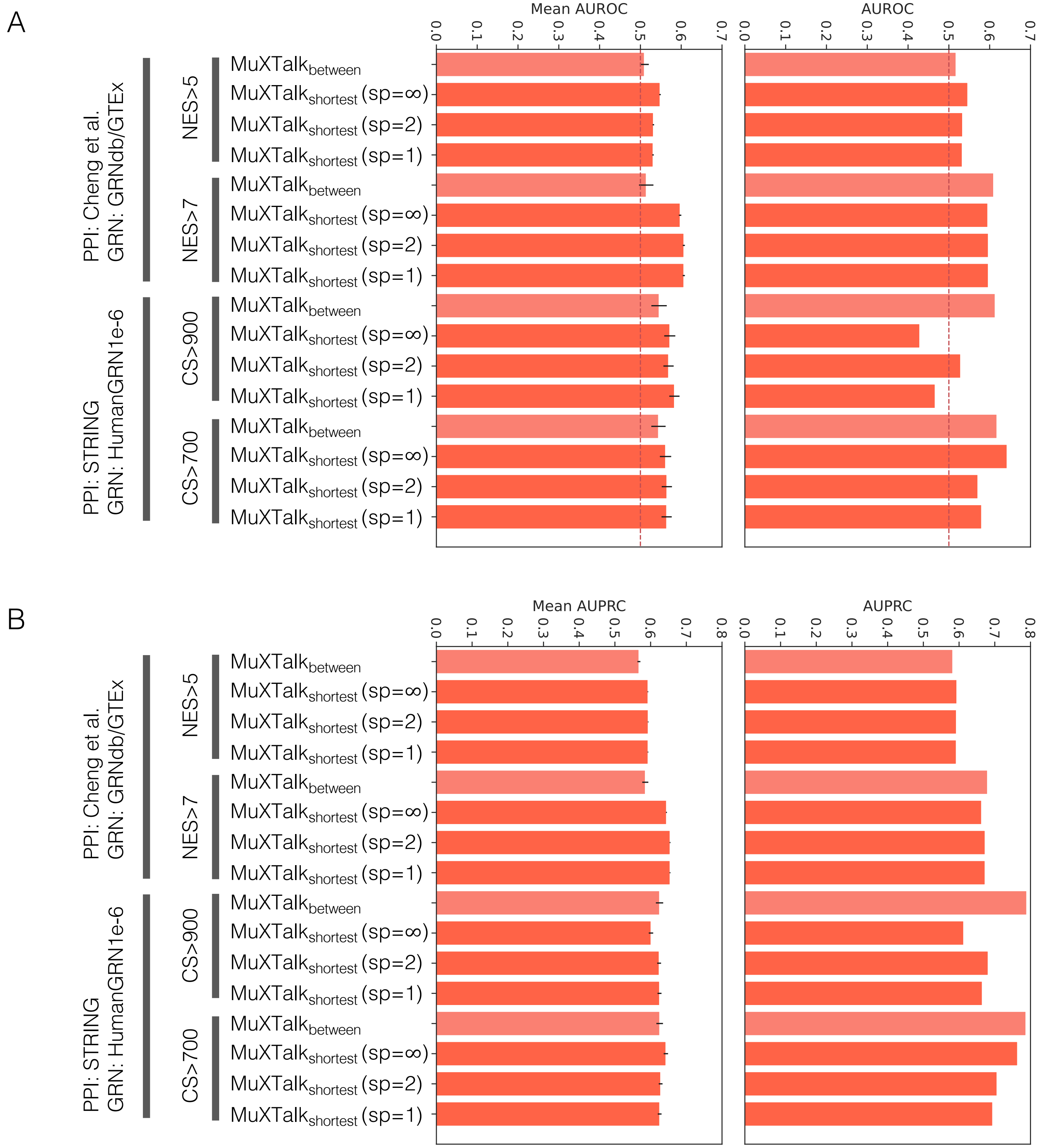

Supplementary Figure 10: Area under the receiver operating characteristic (AUROC) **(A)** and precision-recall curves (AUPRC) **(B)** for MuXTalk for the stochastic (left) and deterministic (right) versions of the benchmark, for multilayer networks withdifferent PPI and GRN layers. Error bars indicate the standard deviation. GRNdb network was constructed by taking the union of tissue-specific GTEx edges in the GRNdb database (http://www.grndb.com/). NES: Normalized Enrichment Scores, used as edge weights in the GRNdb GTEx networks. CS: Confidence Scores used as weights in the STRING PPI network. Cheng et al. and HumanGRN1e-6 denote the PPI and GRN networks, respectively, used in the original analyses.

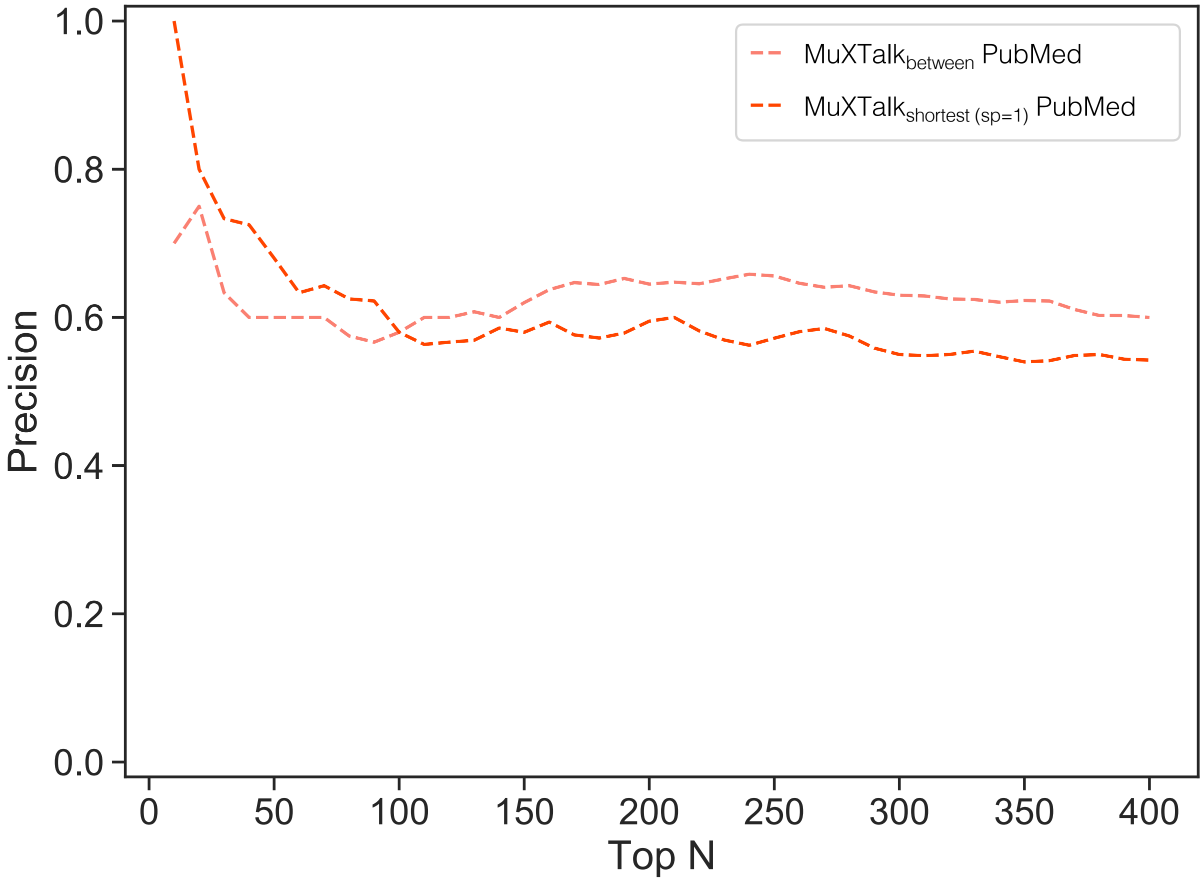

Supplementary Figure 11: Precision-rank plot for the top 400 inferences in the discovery set of pathways for the PubMed query only.

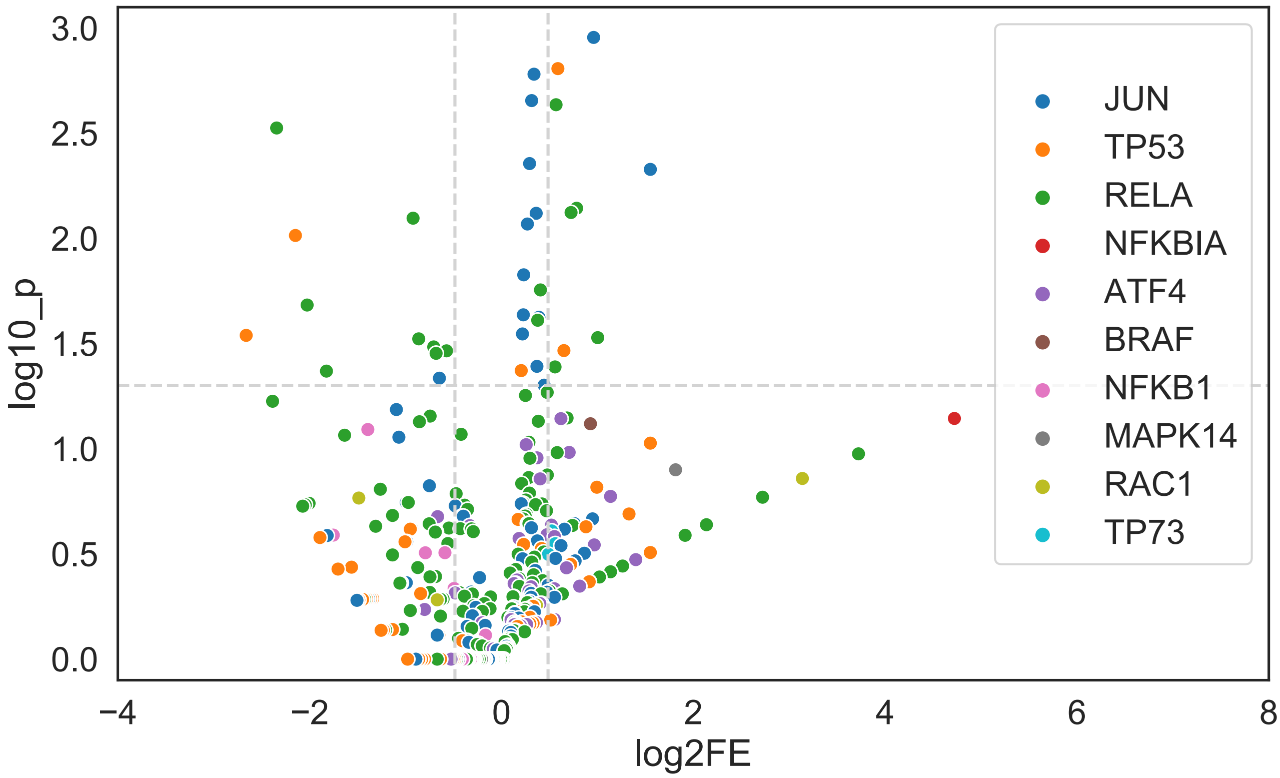

Supplementary Figure 12: The enrichment of ChIP-seq peaks in the TGF-beta signaling pathway corresponding to transcription factors in the neurotrophin signaling pathway (shown in the figure legend). ChIP-seq TF regulatory elements data was obtained from ChIP-Atlas (https://chip-atlas.org/). The volcano plot shows the enrichment log_{10}(p–values) against the log_2 (fold-enrichment) of ChIP-seq peaks. Vertical dashed lines correspond to a fold-enrichment of |1.4|. Horizontal dashed line corresponds to p=0.05.

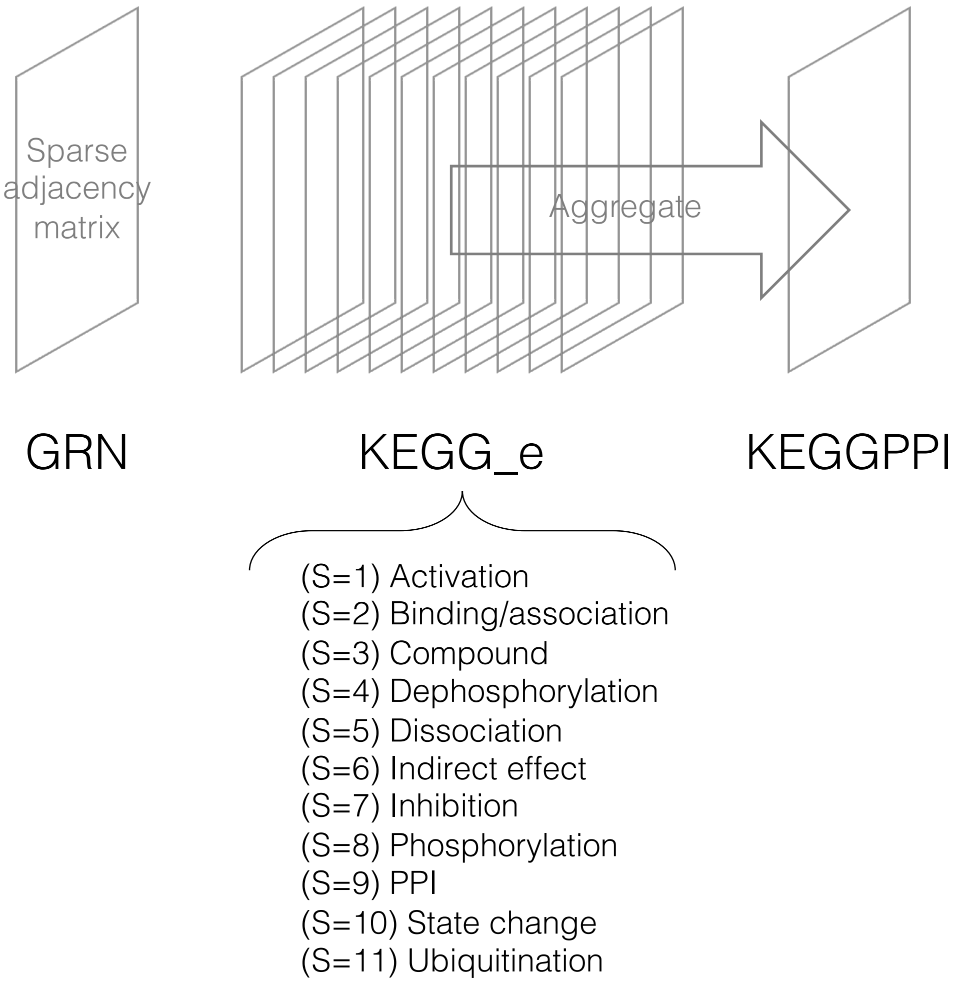

Supplementary Figure 13: Schematic showing the sparse adjacency matrices used in MuXTalk. For each type of sparse matrices, we generated ensembles (N=500) of randomized versions on which to calculate z-scores and empirical p-values.

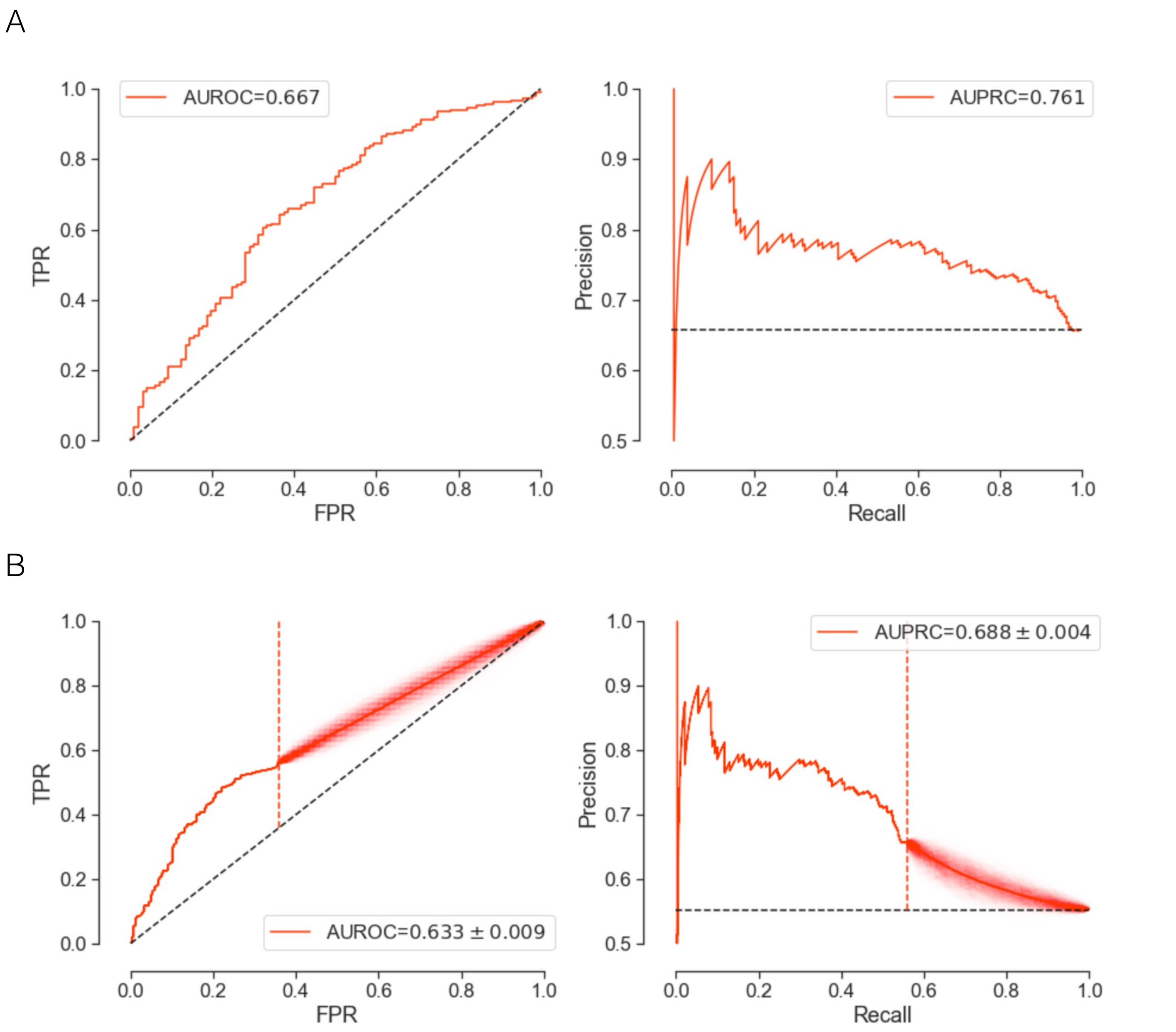

Supplementary Figure 14: Examples showing the deterministic **(A)** and stochastic **(B)** versions of the benchmark.

Supplementary Table Legends

Supplementary Table 1: Properties of the KEGG pathways used in the construction of the KEGG signaling network. Columns are: # of Nodes; # of Edges; Network density; Included in the benchmark? (Yes/No).

Supplementary Table 2: Properties of the gene regulatory networks (GRNs) used as the regulatory network layer. Columns are: # of transcription factors (TFs); # of TF targets; # of Nodes; # of Edges; Network density.

Supplementary Table 3: Allocation plan for the randomized networks showing the breakdown of the randomized networks into which analyses they were used for.

Supplementary Table 4: Two-sided Mann-Whitney U p-values comparing the AUROC **(A)** and AUPRC **(B)** values of MuXTalk and other methods.

Supplementary Table 5: Area under the receiver operating characteristic (AUROC) and precision-recall curves (AUPRC) metrics of the leave-one-layer-out cross-validation (for HumanGRN1e-4). Detected AUC values refer to the deterministic benchmark whereas mean and standard deviations belong to the stochastic benchmark. Row names indicate the signaling layer that was excluded when predicting crosstalk.

Supplementary Table 6: The manually curated list of keywords for each signaling pathway that were used when querying PubMed.

Supplementary Table 7: Pubmed query results for the top $\text{MuXTalk}_{\text{shortest}}$(sp=1) crosstalk predictions. “Counts” refer to the number of publications returned by the PubMed Query. “in_XTalkDB_Gold” denotes whether the crosstalk between Pathway A and B is identified in the XTalkDB Benchmark dataset. “B-->A” means that crosstalk from Pathway B to Pathway A (but not from Pathway A to Pathway B) was in XTalkDB benchmark data. “Neither” means the crosstalk was not identified in XTalkDB in either direction.

Supplementary Table 8: Pubmed query results for the top $\text{MuXTalk}_{\text{between}}$ crosstalk predictions. “Counts” refer to the number of publications returned by the PubMed Query. “in_XTalkDB_Gold” denotes whether the crosstalk between Pathway A and B is identified in the XTalkDB Benchmark dataset. “B-->A” means that crosstalk from Pathway B to Pathway A (but not from Pathway A to Pathway B) was in XTalkDB benchmark data. “Neither” means the crosstalk was not identified in XTalkDB in either direction.

Legends for Supplementary Figures 5-7 (provided as separate pdf files)

Supplementary Figure 5: Multilink profiles of edges within KEGG pathways with a GRN layer of p-value threshold ($p<10-4$).

Supplementary Figure 6: Multilink profiles of edges within KEGG pathways with a GRN layer of p-value threshold ($p<10-5$).

Supplementary Figure 7: Multilink profiles of edges within KEGG pathways with a GRN layer of p-value threshold ($p<10-6$). In **Supp Figs. 5-7**, multilink types are represented by numerical indicators where the first one or two digits represent the signaling edge type and the last digit represents the regulatory interaction direction. Red triangles pointing up and blue triangles pointing down indicate statistically over-represented ($z>0$; emp. $p\leq0.05$) and under-represented ($z<0$; emp. $p\leq0.05$) multilink types, respectively. Black circles denote statistically insignificant multilink types (emp. $p>0.05$).

Supplementary File Legends

Supplementary File 1: Results of the PubMed query-guided manual screening of the 50 top-ranked pathways pairs by $\text{MuXTalk}_{\text{between}}$ in the Discovery analysis. ”a2b” indicates crosstalk from the first pathway to the second pathway; ”b2a” indicates crosstalk from the second pathway to the first pathway. “From PubMed Query” indicates whether the recorded PubMed ID (PMID) is returned by the automated PubMed query or not.

Supplementary File 2: Results of the PubMed query-guided manual screening of the 50 top-ranked pathways pairs by $\text{MuXTalk}_{\text{shortest}}$(sp=1) in the Discovery analysis. ”a2b” indicates crosstalk from the first pathway to the second pathway; ”b2a” indicates crosstalk from the second pathway to the first pathway. “From PubMed Query” indicates whether the recorded PubMed ID (PMID) is returned by the automated PubMed query or not.

Supplementary Table 1

KEGG Signaling Pathway	n_nodes	n_edges	density	benchmark
Adherens junction	68	207	0.04543459	Y
Adipocytokine signaling pathway	61	220	0.06010929	Y
Adrenergic signaling in cardiomyocytes	150	880	0.0393736	N
AGE-RAGE signaling pathway in diabetic complications	69	234	0.04987212	N
AMPK signaling pathway	86	280	0.03830369	N
Apelin signaling pathway	109	804	0.06829766	N
Apoptosis	121	301	0.02073003	Y
B cell receptor signaling pathway	77	201	0.03434723	N
C-type lectin receptor signaling pathway	85	188	0.02633053	N
Calcium signaling pathway	152	872	0.03799233	N
cAMP signaling pathway	192	956	0.02606894	N
Cell adhesion molecules (CAMs)	130	634	0.03780561	N
cGMP-PKG signaling pathway	147	383	0.01784549	N
Chemokine signaling pathway	187	1907	0.05482721	N
Cytokine-cytokine receptor interaction	196	257	0.00672423	N
ECM-receptor interaction	77	210	0.03588517	N
Epithelial cell signaling in Helicobacter pylori infection	31	40	0.04301075	N
ErbB signaling pathway	83	212	0.03114899	Y
Estrogen signaling pathway	88	231	0.03017241	Y
Fc epsilon RI signaling pathway	58	175	0.05293406	N
FoxO signaling pathway	81	262	0.0404321	N
Glucagon signaling pathway	76	406	0.07122807	N
GnRH signaling pathway	86	310	0.04240766	Y
Hedgehog signaling pathway	47	196	0.0906568	Y
HIF-1 signaling pathway	61	149	0.04071038	Y
Hippo signaling pathway	135	676	0.03736871	Y
Insulin secretion	50	87	0.0355102	Y
Insulin signaling pathway	127	436	0.02724659	Y
JAK-STAT signaling pathway	140	3197	0.16428571	Y
MAPK signaling pathway	294	2191	0.02543475	Y
Melanogenesis	99	446	0.0459699	Y
mTOR signaling pathway	133	398	0.02267031	Y
Neuroactive ligand-receptor interaction	190	228	0.00634921	N
Neurotrophin signaling pathway	115	477	0.03638444	Y
NF-kappa B signaling pathway	65	102	0.02451923	Y
NOD-like receptor signaling pathway	148	335	0.01539805	N
Notch signaling pathway	47	190	0.08788159	Y
Oxytocin signaling pathway	132	396	0.02290076	N
p53 signaling pathway	35	56	0.04705882	N
Phosphatidylinositol signaling system	86	2747	0.37578659	N
Phospholipase D signaling pathway	112	450	0.03619691	N
PI3K-Akt signaling pathway	265	2936	0.04196684	N
PPAR signaling pathway	6	24	0.8	N
Prolactin signaling pathway	59	127	0.0371128	Y
Rap1 signaling pathway	204	1227	0.02962909	N
Ras signaling pathway	226	1586	0.03118977	N
Relaxin signaling pathway	109	573	0.04867482	N
Retrograde endocannabinoid signaling	103	672	0.06396345	N
RIG-I-like receptor signaling pathway	47	85	0.03931545	N
Signaling pathways regulating pluripotency of stem cells	98	429	0.04512939	N
Sphingolipid signaling pathway	94	261	0.02985587	N
T cell receptor signaling pathway	94	262	0.02997026	N
TGF-beta signaling pathway	86	302	0.04131327	Y
Thyroid hormone signaling pathway	82	382	0.0575128	N
Thyroid hormone synthesis	44	103	0.05443975	Y
TNF signaling pathway	72	143	0.0279734	Y
Toll-like receptor signaling pathway	69	136	0.02898551	Y
VEGF signaling pathway	58	227	0.06866304	Y
Viral protein interaction with cytokine and cytokine receptor	90	101	0.01260924	N
Wnt signaling pathway	146	1119	0.05285782	Y

Supplementary Table 2

GRN	# TFs	# Targets	# Nodes	# Edges	Density
10e-6	468	10,063	10,245	34,643	0.00066
10e-5	605	11,513	11,708	348,664	0.00509
10e-4	632	11,517	11,716	1,969,966	0.02870

Supplementary Table 3

Analysis	Randomized multilayer ensemble index	Notes
Overall multilink statistics	0-99	100 randomizations of the entire multilayer network
Pathway-specific multilink statistics	100-6199	61x100 randomizations of the entire multilayer network
$MuXTalk_{between}$ benchmark	6200-66199	600 (# of pathway pairs in the benchmark)x100 randomizations of the entire multilayer network
$MuXTalk_{shortest}$ benchmark	66200-126199	600 (# of pathway pairs in the benchmark)x100 randomizations of the entire multilayer network

Supplementary Table 4

A

	XTalk (k=1)	XTalk (k=2)	XTalk (k=4)	XTalk (k=6)	XTalk (k=8)	XTalk (k=10)	XTalk (k=20)	XTalk (k=50)	XTalk (k=100)	Node overlap	Edge overlap	Direct edges
MuXTalk$_{between}$	4.55e-50	2.03e-48	2.19e-76	2.11e-103	8.81e-140	1.10e-190	8.51e-239	< 1e-217	< 1e-217	< 1e-217	< 1e-217	< 1e-217
MuXTalk$_{shortest}$(sp=1)	< 1e-217	< 1e-217	< 1e-217	< 1e-217	< 1e-217	< 1e-217	< 1e-217	< 1e-217	< 1e-217	< 1e-217	< 1e-217	< 1e-217
MuXTalk$_{shortest}$(sp=2)	< 1e-217	< 1e-217	< 1e-217	< 1e-217	< 1e-217	< 1e-217	< 1e-217	< 1e-217	< 1e-217	< 1e-217	< 1e-217	< 1e-217
MuXTalk$_{shortest}$(sp=∞)	< 1e-217	< 1e-217	< 1e-217	< 1e-217	< 1e-217	< 1e-217	< 1e-217	< 1e-217	< 1e-217	< 1e-217	< 1e-217	< 1e-217

B

	XTalk (k=1)	XTalk (k=2)	XTalk (k=4)	XTalk (k=6)	XTalk (k=8)	XTalk (k=10)	XTalk (k=20)	XTalk (k=50)	XTalk (k=100)	Node overlap	Edge overlap	Direct edges
MuXTalk$_{between}$	< 1e-217	< 1e-217	< 1e-217	< 1e-217	< 1e-217	< 1e-217	< 1e-217	< 1e-217	< 1e-217	< 1e-217	< 1e-217	< 1e-217
MuXTalk$_{shortest}$(sp=1)	< 1e-217	< 1e-217	< 1e-217	< 1e-217	< 1e-217	< 1e-217	< 1e-217	< 1e-217	< 1e-217	< 1e-217	< 1e-217	< 1e-217
MuXTalk$_{shortest}$(sp=2)	< 1e-217	< 1e-217	< 1e-217	< 1e-217	< 1e-217	< 1e-217	< 1e-217	< 1e-217	< 1e-217	< 1e-217	< 1e-217	< 1e-217
MuXTalk$_{shortest}$(sp=∞)	< 1e-217	< 1e-217	< 1e-217	< 1e-217	< 1e-217	< 1e-217	< 1e-217	< 1e-217	< 1e-217	< 1e-217	< 1e-217	< 1e-217

Supplementary Table 5

excluded layer	method	GRN	sp thres hold	detected AUROC	detected AUPRC	AUROC mean	AUPRC mean	AUROC std	AUPRC std
activation	MuXTalk_between	HumanGRN10e4		0.54950591	0.62299907	0.58919935	0.61267119	0.00303584	0.00121667
binding/association	MuXTalk_between	HumanGRN10e4		0.53747002	0.61768406	0.58634287	0.60938592	0.00277192	0.00108539
compound	MuXTalk_between	HumanGRN10e4		0.54119171	0.62001527	0.59000228	0.61161926	0.0026546	0.00112211
dephosphorylation	MuXTalk_between	HumanGRN10e4		0.54119171	0.62001527	0.59011953	0.61165215	0.00274629	0.0011206
dissociation	MuXTalk_between	HumanGRN10e4		0.54119171	0.62001527	0.59020913	0.61159918	0.00269885	0.00115813
indirect effect	MuXTalk_between	HumanGRN10e4		0.54085862	0.61943523	0.58987403	0.61114776	0.00273112	0.00110397
inhibition	MuXTalk_between	HumanGRN10e4		0.54179434	0.61992453	0.58881745	0.611345	0.00265208	0.00112743
phosphorylation	MuXTalk_between	HumanGRN10e4		0.54135069	0.62036724	0.59393549	0.61208864	0.00268664	0.00116836
state change	MuXTalk_between	HumanGRN10e4		0.54119171	0.62001527	0.59011665	0.61167058	0.00269942	0.00114384
ubiquitination	MuXTalk_between	HumanGRN10e4		0.54119171	0.62001527	0.59028171	0.61170541	0.00266083	0.00110731
activation	MuXTalk_shortest	HumanGRN10e4	1	0.53179557	0.58604054	0.53368097	0.58514756	0.00185061	0.00083303
binding/association	MuXTalk_shortest	HumanGRN10e4	1	0.52342831	0.5752258	0.52549972	0.5743071	0.00185282	0.00083402
compound	MuXTalk_shortest	HumanGRN10e4	1	0.52441203	0.58914339	0.52642123	0.58820767	0.00185299	0.0008341
dephosphorylation	MuXTalk_shortest	HumanGRN10e4	1	0.53270014	0.59652324	0.53464987	0.59556689	0.00185294	0.00083408
dissociation	MuXTalk_shortest	HumanGRN10e4	1	0.52323609	0.58751805	0.52524208	0.58659224	0.00185282	0.00083402
indirect effect	MuXTalk_shortest	HumanGRN10e4	1	0.52273858	0.58973407	0.52474421	0.58880324	0.00185275	0.00083399
inhibition	MuXTalk_shortest	HumanGRN10e4	1	0.54295568	0.59412684	0.544766	0.59320944	0.00185061	0.00083303
phosphorylation	MuXTalk_shortest	HumanGRN10e4	1	0.525	0.58846956	0.52696077	0.58755589	0.00185192	0.00083362
state change	MuXTalk_shortest	HumanGRN10e4	1	0.52873134	0.5886047	0.53070037	0.58767561	0.00185282	0.00083402
ubiquitination	MuXTalk_shortest	HumanGRN10e4	1	0.52441203	0.58914339	0.52643976	0.58819933	0.00185312	0.00083416
activation	MuXTalk_shortest	HumanGRN10e4	2	0.52928492	0.5821675	0.52928492	0.5821675	2.22E-16	2.22E-16
binding/association	MuXTalk_shortest	HumanGRN10e4	2	0.5220746	0.57120497	0.5220746	0.57120497	1.11E-16	0
compound	MuXTalk_shortest	HumanGRN10e4	2	0.52477004	0.58568722	0.52477004	0.58568722	1.11E-16	0
dephosphorylation	MuXTalk_shortest	HumanGRN10e4	2	0.53375487	0.59153586	0.53375487	0.59153586	1.11E-16	0
dissociation	MuXTalk_shortest	HumanGRN10e4	2	0.52357956	0.58408409	0.52357956	0.58408409	1.11E-16	1.11E-16
indirect effect	MuXTalk_shortest	HumanGRN10e4	2	0.52304047	0.58733801	0.52304047	0.58733801	2.22E-16	1.11E-16
inhibition	MuXTalk_shortest	HumanGRN10e4	2	0.54264985	0.5947754	0.54264985	0.5947754	1.11E-16	1.11E-16
phosphorylation	MuXTalk_shortest	HumanGRN10e4	2	0.52828536	0.58733267	0.52828536	0.58733267	0	1.11E-16
state change	MuXTalk_shortest	HumanGRN10e4	2	0.52726333	0.58364687	0.52726333	0.58364687	2.22E-16	1.11E-16
ubiquitination	MuXTalk_shortest	HumanGRN10e4	2	0.52477004	0.58568722	0.52477004	0.58568722	1.11E-16	0
activation	MuXTalk_shortest	HumanGRN10e4	None	0.52682532	0.58188392	0.52682532	0.58188392	1.11E-16	1.11E-16
binding/association	MuXTalk_shortest	HumanGRN10e4	None	0.51992947	0.57149086	0.51992947	0.57149086	2.22E-16	0
compound	MuXTalk_shortest	HumanGRN10e4	None	0.52293939	0.58573563	0.52293939	0.58573563	2.22E-16	0
dephosphorylation	MuXTalk_shortest	HumanGRN10e4	None	0.53186806	0.59161223	0.53186806	0.59161223	1.11E-16	0
dissociation	MuXTalk_shortest	HumanGRN10e4	None	0.52176013	0.58414951	0.52176013	0.58414951	0	2.22E-16
indirect effect	MuXTalk_shortest	HumanGRN10e4	None	0.52118734	0.58766131	0.52118734	0.58766131	0	0
inhibition	MuXTalk_shortest	HumanGRN10e4	None	0.54430081	0.59299756	0.54430081	0.59299756	1.11E-16	1.11E-16
phosphorylation	MuXTalk_shortest	HumanGRN10e4	None	0.52640978	0.58709797	0.52640978	0.58709797	1.11E-16	1.11E-16
state change	MuXTalk_shortest	HumanGRN10e4	None	0.52524175	0.58272404	0.52524175	0.58272404	0	0
ubiquitination	MuXTalk_shortest	HumanGRN10e4	None	0.52293939	0.58573563	0.52293939	0.58573563	2.22E-16	0

Supplementary Table 5 (cont'd)

excluded layer	method	GRN	sp threshold	detected AUROC	detected AUPRC	AUROC mean	AUPRC mean	AUROC std	AUPRC std
activation	MuXTalk_between	HumanGRN10e5		0.58604232	0.67370503	0.57499815	0.62761909	0.00909387	0.00356138
binding/association	MuXTalk_between	HumanGRN10e5		0.57333738	0.65919927	0.57724915	0.62188103	0.00910562	0.00340187
compound	MuXTalk_between	HumanGRN10e5		0.57333738	0.65919927	0.57678635	0.62184596	0.0091153	0.00340017
dephosphorylation	MuXTalk_between	HumanGRN10e5		0.57333738	0.65919927	0.57756219	0.62215117	0.00880638	0.00335
dissociation	MuXTalk_between	HumanGRN10e5		0.57333738	0.65919927	0.57629767	0.62166352	0.00922748	0.00343511
indirect effect	MuXTalk_between	HumanGRN10e5		0.57333738	0.65919927	0.57721318	0.62196038	0.00888567	0.00339968
inhibition	MuXTalk_between	HumanGRN10e5		0.58472516	0.6661722	0.57139175	0.62269046	0.00909157	0.00343629
phosphorylation	MuXTalk_between	HumanGRN10e5		0.56383088	0.65840308	0.57110294	0.62023186	0.00906368	0.00351094
state change	MuXTalk_between	HumanGRN10e5		0.57333738	0.65919927	0.57676535	0.62180045	0.00885483	0.00330567
ubiquitination	MuXTalk_between	HumanGRN10e5		0.57333738	0.65919927	0.57660936	0.6217726	0.00873965	0.00330349
activation	MuXTalk_shortest	HumanGRN10e5	1	0.62489316	0.65945416	0.62581482	0.65650413	0.00179746	0.00083366
binding/association	MuXTalk_shortest	HumanGRN10e5	1	0.62686372	0.65459888	0.62773384	0.65172367	0.00179983	0.00083885
compound	MuXTalk_shortest	HumanGRN10e5	1	0.62686372	0.65459888	0.62781356	0.65168651	0.00180682	0.00084272
dephosphorylation	MuXTalk_shortest	HumanGRN10e5	1	0.62686372	0.65459888	0.62766433	0.65175819	0.00179085	0.00083366
dissociation	MuXTalk_shortest	HumanGRN10e5	1	0.62623457	0.65256118	0.62712981	0.64973206	0.00179934	0.00083828
indirect effect	MuXTalk_shortest	HumanGRN10e5	1	0.62686372	0.65459888	0.62774971	0.65171725	0.00180534	0.00083867
inhibition	MuXTalk_shortest	HumanGRN10e5	1	0.62941595	0.64660256	0.62650583	0.64556785	0.00180072	0.00083895
phosphorylation	MuXTalk_shortest	HumanGRN10e5	1	0.62757799	0.65663084	0.62663332	0.65333754	0.00180912	0.00084423
state change	MuXTalk_shortest	HumanGRN10e5	1	0.62686372	0.65459888	0.62774948	0.65171459	0.00180274	0.00083953
ubiquitination	MuXTalk_shortest	HumanGRN10e5	1	0.62686372	0.65459888	0.6277464	0.65171903	0.00180692	0.00083801
activation	MuXTalk_shortest	HumanGRN10e5	2	0.58931589	0.62109541	0.58860246	0.61909658	0.00181592	0.00083083
binding/association	MuXTalk_shortest	HumanGRN10e5	2	0.59665471	0.61912377	0.59571507	0.61715795	0.00181613	0.00083149
compound	MuXTalk_shortest	HumanGRN10e5	2	0.59665471	0.61912377	0.59575531	0.61713946	0.00180882	0.00082907
dephosphorylation	MuXTalk_shortest	HumanGRN10e5	2	0.59665471	0.61912377	0.59561571	0.61720317	0.00182749	0.00083633
dissociation	MuXTalk_shortest	HumanGRN10e5	2	0.59543929	0.6170877	0.59450971	0.61516427	0.0018218	0.00083244
indirect effect	MuXTalk_shortest	HumanGRN10e5	2	0.59665471	0.61912377	0.59568658	0.61717158	0.0018217	0.00083278
inhibition	MuXTalk_shortest	HumanGRN10e5	2	0.58995254	0.61745216	0.5892942	0.61547497	0.00180285	0.00082568
phosphorylation	MuXTalk_shortest	HumanGRN10e5	2	0.59450168	0.61466978	0.59354114	0.61280968	0.00182553	0.00083593
state change	MuXTalk_shortest	HumanGRN10e5	2	0.59665471	0.61912377	0.5956132	0.61720513	0.00182947	0.00083605
ubiquitination	MuXTalk_shortest	HumanGRN10e5	2	0.59665471	0.61912377	0.59563483	0.61719497	0.00182615	0.00083555
activation	MuXTalk_shortest	HumanGRN10e5	None	0.58703123	0.61853436	0.58514013	0.61643582	0.00175385	0.00080011
binding/association	MuXTalk_shortest	HumanGRN10e5	None	0.5947576	0.61699905	0.59250764	0.61499596	0.00179598	0.00081903
compound	MuXTalk_shortest	HumanGRN10e5	None	0.5947576	0.61699905	0.59258554	0.61495835	0.00177401	0.00081078
dephosphorylation	MuXTalk_shortest	HumanGRN10e5	None	0.5947576	0.61699905	0.59256417	0.61496858	0.00177961	0.0008134
dissociation	MuXTalk_shortest	HumanGRN10e5	None	0.59348909	0.6149415	0.59141522	0.61290145	0.00175522	0.00080217
indirect effect	MuXTalk_shortest	HumanGRN10e5	None	0.5947576	0.61699905	0.59254184	0.61497857	0.00178696	0.00081538
inhibition	MuXTalk_shortest	HumanGRN10e5	None	0.58933761	0.61630298	0.587323	0.61426643	0.00177072	0.00080881
phosphorylation	MuXTalk_shortest	HumanGRN10e5	None	0.59227824	0.61244401	0.59015233	0.61048045	0.00178014	0.00081261
state change	MuXTalk_shortest	HumanGRN10e5	None	0.5947576	0.61699905	0.59259857	0.61495382	0.00177107	0.00080944
ubiquitination	MuXTalk_shortest	HumanGRN10e5	None	0.5947576	0.61699905	0.59252552	0.61498487	0.00179077	0.00081685

Supplementary Table 5 (cont'd)

excluded layer	method	GRN	sp thres hold	detected AUROC	detected AUPRC	AUROC mean	AUPRC mean	AUROC std	AUPRC std
activation	MuXTalk_between	HumanGRN10e6		0.55943438	0.74028029	0.55180155	0.62178775	0.01726323	0.00910809
binding/association	MuXTalk_between	HumanGRN10e6		0.55943438	0.74028029	0.55108349	0.62141479	0.0174445	0.00916585
compound	MuXTalk_between	HumanGRN10e6		0.55943438	0.74028029	0.55144558	0.6216602	0.01789201	0.0094294
dephosphorylation	MuXTalk_between	HumanGRN10e6		0.55943438	0.74028029	0.55271231	0.62239969	0.01775745	0.00942063
dissociation	MuXTalk_between	HumanGRN10e6		0.55943438	0.74028029	0.5530464	0.62249334	0.01782365	0.00942053
indirect effect	MuXTalk_between	HumanGRN10e6		0.55943438	0.74028029	0.55229697	0.62219314	0.0181133	0.00975105
inhibition	MuXTalk_between	HumanGRN10e6		0.55943438	0.74028029	0.55287304	0.62247082	0.01713974	0.00912783
phosphorylation	MuXTalk_between	HumanGRN10e6		0.55943438	0.74028029	0.55259238	0.62216967	0.01803552	0.00969788
state change	MuXTalk_between	HumanGRN10e6		0.55943438	0.74028029	0.55201207	0.62197775	0.01701221	0.00908082
ubiquitination	MuXTalk_between	HumanGRN10e6		0.55943438	0.74028029	0.55196234	0.62206078	0.01728799	0.00922549
activation	MuXTalk_shortest	HumanGRN10e6	1	0.62417249	0.75314373	0.6349744	0.68601234	0.00935545	0.0038467
binding/association	MuXTalk_shortest	HumanGRN10e6	1	0.62508351	0.75553338	0.63498158	0.68723071	0.00991993	0.00391855
compound	MuXTalk_shortest	HumanGRN10e6	1	0.62508351	0.75553338	0.63455997	0.68702659	0.00993008	0.00395688
dephosphorylation	MuXTalk_shortest	HumanGRN10e6	1	0.62508351	0.75553338	0.63519751	0.68739068	0.00956339	0.00385504
dissociation	MuXTalk_shortest	HumanGRN10e6	1	0.62508351	0.75553338	0.63517162	0.68726712	0.00957565	0.00379396
indirect effect	MuXTalk_shortest	HumanGRN10e6	1	0.62508351	0.75553338	0.63511811	0.68723742	0.00931195	0.00365041
inhibition	MuXTalk_shortest	HumanGRN10e6	1	0.62508351	0.75553338	0.63543239	0.68742615	0.00927224	0.00365281
phosphorylation	MuXTalk_shortest	HumanGRN10e6	1	0.62508351	0.75553338	0.63549586	0.6873787	0.00926837	0.00369531
state change	MuXTalk_shortest	HumanGRN10e6	1	0.62508351	0.75553338	0.63519205	0.68722046	0.00932362	0.00374723
ubiquitination	MuXTalk_shortest	HumanGRN10e6	1	0.62508351	0.75553338	0.63551665	0.68729927	0.00935668	0.00374024
activation	MuXTalk_shortest	HumanGRN10e6	2	0.59764262	0.72856951	0.63089672	0.67698717	0.0079286	0.00298162
binding/association	MuXTalk_shortest	HumanGRN10e6	2	0.59844413	0.73049673	0.63192179	0.67834725	0.00819755	0.0031673
compound	MuXTalk_shortest	HumanGRN10e6	2	0.59844413	0.73049673	0.63168774	0.67833602	0.00813462	0.0031477
dephosphorylation	MuXTalk_shortest	HumanGRN10e6	2	0.59844413	0.73049673	0.63164845	0.67837735	0.00794723	0.00297975
dissociation	MuXTalk_shortest	HumanGRN10e6	2	0.59844413	0.73049673	0.63121236	0.67822279	0.0081369	0.00313721
indirect effect	MuXTalk_shortest	HumanGRN10e6	2	0.59844413	0.73049673	0.6318803	0.67844663	0.00817041	0.00318789
inhibition	MuXTalk_shortest	HumanGRN10e6	2	0.59844413	0.73049673	0.6314855	0.67829206	0.00807091	0.0031508
phosphorylation	MuXTalk_shortest	HumanGRN10e6	2	0.59844413	0.73049673	0.63125633	0.67822029	0.00812802	0.00307864
state change	MuXTalk_shortest	HumanGRN10e6	2	0.59844413	0.73049673	0.63109651	0.67810771	0.00833929	0.00312844
ubiquitination	MuXTalk_shortest	HumanGRN10e6	2	0.59844413	0.73049673	0.6313976	0.67819298	0.00789983	0.00307841
activation	MuXTalk_shortest	HumanGRN10e6	None	0.59787836	0.72868815	0.63078065	0.67698817	0.00823983	0.00315398
binding/association	MuXTalk_shortest	HumanGRN10e6	None	0.59867987	0.73061537	0.63164845	0.67838523	0.00821988	0.00312997
compound	MuXTalk_shortest	HumanGRN10e6	None	0.59867987	0.73061537	0.63156604	0.67832757	0.00820395	0.00311636
dephosphorylation	MuXTalk_shortest	HumanGRN10e6	None	0.59867987	0.73061537	0.63153127	0.67830508	0.00795267	0.00309819
dissociation	MuXTalk_shortest	HumanGRN10e6	None	0.59867987	0.73061537	0.63143297	0.67834727	0.008186	0.00313427
indirect effect	MuXTalk_shortest	HumanGRN10e6	None	0.59867987	0.73061537	0.63126972	0.67819793	0.00808133	0.00313106
inhibition	MuXTalk_shortest	HumanGRN10e6	None	0.59867987	0.73061537	0.63172988	0.67843494	0.00818823	0.00315064
phosphorylation	MuXTalk_shortest	HumanGRN10e6	None	0.59867987	0.73061537	0.63122067	0.67819206	0.00791772	0.00310188
state change	MuXTalk_shortest	HumanGRN10e6	None	0.59867987	0.73061537	0.63147022	0.67838782	0.00818447	0.00313837
ubiquitination	MuXTalk_shortest	HumanGRN10e6	None	0.59867987	0.73061537	0.63174339	0.67838851	0.00778562	0.0030185

Supplementary Table 6

Pathway Name	Keywords	Query
AGE-RAGE signaling pathway in diabetic complications	AGE-RAGE diabetic	AGE-RAGE AND diabetic
AMPK signaling pathway	AMPK	AMPK
Adherens junction	Adherens	Adherens
Adipocytokine signaling pathway	Adipocytokine	Adipocytokine
Adrenergic signaling in cardiomyocytes	Adrenergic cardiomyocytes	Adrenergic AND cardiomyocytes
Apelin signaling pathway	Apelin	Apelin
Apoptosis	Apoptosis	Apoptosis
B cell receptor signaling pathway	B cell	"B cell"
C-type lectin receptor signaling pathway	C-type lectin	"C-type lectin"
Calcium signaling pathway	Calcium	Calcium
Cell adhesion molecules (CAMs)	Cell adhesion CAM	"Cell adhesion" OR CAM
Chemokine signaling pathway	Chemokine	Chemokine
Cytokine-cytokine receptor interaction	Cytokine	Cytokine
ECM-receptor interaction	ECM	ECM
Epithelial cell signaling in Helicobacter pylori infection	Epithelial Helicobacter	Epithelial AND Helicobacter
ErbB signaling pathway	ErbB	ErbB
Estrogen signaling pathway	Estrogen	Estrogen
Fc epsilon RI signaling pathway	Fc epsilon RI	"Fc epsilon" OR "Fc epsilon RI" OR FCER1
FoxO signaling pathway	FoxO	FoxO
Glucagon signaling pathway	Glucagon	Glucagon
GnRH signaling pathway	GnRH	GnRH
HIF-1 signaling pathway	HIF-1	HIF-1
Hedgehog signaling pathway	Hedgehog	Hedgehog
Hippo signaling pathway	Hippo	Hippo
Insulin secretion	Insulin secretion	Insulin AND secretion
Insulin signaling pathway	Insulin	Insulin
JAK-STAT signaling pathway	JAK-STAT	JAK-STAT
MAPK signaling pathway	MAPK	MAPK
Melanogenesis	Melanogenesis	Melanogenesis
NF-kappa B signaling pathway	NF-kappa B	"NF-kappa B" OR NF-kb
NOD-like receptor signaling pathway	NOD-like	NOD-like
Neuroactive ligand-receptor interaction	Neuroactive ligand-receptor	Neuroactive AND ligand AND receptor
Neurotrophin signaling pathway	Neurotrophin	Neurotrophin
Notch signaling pathway	Notch	Notch
Oxytocin signaling pathway	Oxytocin	Oxytocin
PI3K-Akt signaling pathway	PI3K-Akt	PI3K-Akt OR PI3K OR Akt
PPAR signaling pathway	PPAR	PPAR
Phosphatidylinositol signaling system	Phosphatidylinositol	Phosphatidylinositol
Phospholipase D signaling pathway	Phospholipase D	Phospholipase AND D
Prolactin signaling pathway	Prolactin	Prolactin
RIG-I-like receptor signaling pathway	RIG-I-like receptor	RIG-I-like OR "RIG-I-like receptor" OR RLR
Rap1 signaling pathway	Rap1	Rap1
Ras signaling pathway	Ras	Ras
Relaxin signaling pathway	Relaxin	Relaxin
Retrograde endocannabinoid signaling	Retrograde endocannabinoid	"Retrograde endocannabinoid"
Signaling pathways regulating pluripotency of stem cells	pluripotency of stem cells	pluripotency OR "stem cells" OR "pluripotency of stem cells"
Sphingolipid signaling pathway	Sphingolipid	Sphingolipid
T cell receptor signaling pathway	T cell	"T cell"
TGF-beta signaling pathway	TGF-beta	TGF-beta OR TGFB OR "Transforming growth factor"
TNF signaling pathway	TNF	TNF OR "tumor necrosis factor"
Thyroid hormone signaling pathway	Thyroid hormone	Thyroid OR "Thyroid hormone"
Thyroid hormone synthesis	Thyroid hormone	Thyroid OR "Thyroid hormone" AND synthesis
Toll-like receptor signaling pathway	Toll-like	Toll-like OR TLR
VEGF signaling pathway	VEGF	VEGF
Viral protein interaction with cytokine and cytokine receptor	Viral cytokine receptor	Viral AND cytokine AND receptor
Wnt signaling pathway	Wnt	Wnt
cAMP signaling pathway	cAMP	cAMP
cGMP-PKG signaling pathway	cGMP-PKG	cGMP OR PKG OR cGMP-PKG
mTOR signaling pathway	mTOR	mTOR
p53 signaling pathway	p53	p53

Supplementary Table 7

Pathway A	Pathway B	MuXTalk_score	Counts	in_XTalkDB_Gold
Neurotrophin signaling pathway	TGF-beta signaling pathway	2106.17937	24	B->A
Notch signaling pathway	Neurotrophin signaling pathway	2034.47137	7	B->A
JAK-STAT signaling pathway	Neurotrophin signaling pathway	2020.89474	5	B->A
Neurotrophin signaling pathway	Estrogen signaling pathway	1179.7	20	B->A
Insulin secretion	Wnt signaling pathway	1089.7	57	B->A
Toll-like receptor signaling pathway	Estrogen signaling pathway	1086.96585	4	B->A
Insulin secretion	Toll-like receptor signaling pathway	1059.7	19	B->A
mTOR signaling pathway	Estrogen signaling pathway	1044.89109	54	B->A
Insulin secretion	MAPK signaling pathway	1042.2159	106	Neither
Insulin secretion	Hippo signaling pathway	1042.2159	4	B->A
Thyroid hormone synthesis	Toll-like receptor signaling pathway	1042.2159	1	Neither
Notch signaling pathway	Thyroid hormone synthesis	1042.2159	3	B->A
Insulin secretion	Notch signaling pathway	1034.47137	15	B->A
GnRH signaling pathway	Wnt signaling pathway	1029.85584	2	B->A
MAPK signaling pathway	Thyroid hormone synthesis	1029.85584	10	B->A
mTOR signaling pathway	TGF-beta signaling pathway	1029.01071	30	B->A
Melanogenesis	Hippo signaling pathway	1026.7071	0	Neither
Apoptosis	NF-kappa B signaling pathway	1026.45449	279	B->A
ErbB signaling pathway	Apoptosis	1024.38378	115	Neither
Hippo signaling pathway	Melanogenesis	1024.38378	0	Neither
VEGF signaling pathway	Estrogen signaling pathway	1023.9539	15	B->A
JAK-STAT signaling pathway	Estrogen signaling pathway	1023.64699	6	B->A
Apoptosis	TGF-beta signaling pathway	1021.7879	195	B->A
Wnt signaling pathway	Prolactin signaling pathway	1019.92188	0	Neither
Insulin secretion	JAK-STAT signaling pathway	1019.39021	17	Neither
Hippo signaling pathway	Thyroid hormone synthesis	1019.39021	0	Neither
Toll-like receptor signaling pathway	Melanogenesis	1019.39021	0	Neither
GnRH signaling pathway	JAK-STAT signaling pathway	1019.39021	0	B->A
Melanogenesis	Toll-like receptor signaling pathway	1019.39021	0	Neither
NF-kappa B signaling pathway	Insulin secretion	1019.39021	41	Neither
Notch signaling pathway	Adipocytokine signaling pathway	1019.39021	11	B->A
Apoptosis	Prolactin signaling pathway	1018.90476	9	B->A
Apoptosis	MAPK signaling pathway	1018.90476	237	B->A
Apoptosis	Estrogen signaling pathway	1017.58705	120	B->A
NF-kappa B signaling pathway	MAPK signaling pathway	1017.2689	99	B->A
Apoptosis	Hippo signaling pathway	1016.00068	44	B->A
Apoptosis	Wnt signaling pathway	1014.98156	169	B->A
TGF-beta signaling pathway	Insulin secretion	1014.54208	58	Neither
Prolactin signaling pathway	Wnt signaling pathway	1014.14057	0	Neither
Notch signaling pathway	Prolactin signaling pathway	1013.77203	1	B->A
ErbB signaling pathway	HIF-1 signaling pathway	1011.6856	6	B->A
Thyroid hormone synthesis	Hippo signaling pathway	1011.6856	0	Neither
ErbB signaling pathway	Insulin secretion	1011.6856	63	Neither
ErbB signaling pathway	Melanogenesis	1011.6856	0	Neither
Insulin secretion	Adherens junction	1008.53548	0	Neither
Melanogenesis	Notch signaling pathway	1008.53548	0	Neither
Insulin signaling pathway	Insulin secretion	1008.53548	1360	Neither
JAK-STAT signaling pathway	ErbB signaling pathway	1008.53548	2	B->A
ErbB signaling pathway	Thyroid hormone synthesis	1008.53548	5	Neither
Thyroid hormone synthesis	ErbB signaling pathway	1008.53548	5	Neither
JAK-STAT signaling pathway	Thyroid hormone synthesis	1008.53548	2	B->A
JAK-STAT signaling pathway	HIF-1 signaling pathway	1008.53548	1	B->A
Adherens junction	Melanogenesis	1008.53548	0	Neither
Hedgehog signaling pathway	GnRH signaling pathway	1008.53548	0	Neither
Apoptosis	GnRH signaling pathway	1008.53548	3	B->A
Adherens junction	Thyroid hormone synthesis	1008.53548	0	Neither
HIF-1 signaling pathway	Apoptosis	1008.53548	44	B->A
NF-kappa B signaling pathway	Melanogenesis	1006.76188	0	Neither
NF-kappa B signaling pathway	Prolactin signaling pathway	1006.76188	4	B->A
Hedgehog signaling pathway	Thyroid hormone synthesis	1006.76188	4	B->A
NF-kappa B signaling pathway	Thyroid hormone synthesis	1006.76188	2	B->A
Melanogenesis	ErbB signaling pathway	1006.76188	0	Neither
Neurotrophin signaling pathway	ErbB signaling pathway	1006.76188	44	Neither
GnRH signaling pathway	Notch signaling pathway	1006.76188	0	Neither
Apoptosis	Adipocytokine signaling pathway	1006.76188	15	B->A
Insulin secretion	Insulin signaling pathway	1006.76188	1360	Neither
Insulin secretion	NF-kappa B signaling pathway	1006.76188	41	Neither

Supplementary Table 7 (cont'd)

Pathway A	Pathway B	MuXTalk_score	Counts	in_XTalkDB_Gold
Wnt signaling pathway	Toll-like receptor signaling pathway	1006.76188	15	B->A
Apoptosis	TNF signaling pathway	1006.76188	298	B->A
Notch signaling pathway	GnRH signaling pathway	1005.60533	0	Neither
Melanogenesis	NF-kappa B signaling pathway	1005.60533	0	Neither
Notch signaling pathway	Melanogenesis	1005.60533	0	Neither
GnRH signaling pathway	Hedgehog signaling pathway	1005.60533	0	Neither
Melanogenesis	Estrogen signaling pathway	1004.53845	0	B->A
JAK-STAT signaling pathway	Wnt signaling pathway	1004.37326	27	Neither
Melanogenesis	TGF-beta signaling pathway	1003.4617	1	B->A
NF-kappa B signaling pathway	ErbB signaling pathway	1003	20	B->A
JAK-STAT signaling pathway	TGF-beta signaling pathway	1003	22	B->A
Toll-like receptor signaling pathway	Thyroid hormone synthesis	1003	1	Neither
GnRH signaling pathway	Prolactin signaling pathway	1003	0	B->A
Apoptosis	ErbB signaling pathway	1003	115	Neither
Melanogenesis	MAPK signaling pathway	1003	1	B->A
Neurotrophin signaling pathway	Prolactin signaling pathway	1003	2	Neither
Prolactin signaling pathway	Neurotrophin signaling pathway	1003	2	Neither
MAPK signaling pathway	Insulin secretion	1003	106	Neither
Insulin secretion	ErbB signaling pathway	1003	63	Neither
Apoptosis	Notch signaling pathway	1003	100	B->A
Insulin secretion	TGF-beta signaling pathway	1003	58	Neither

Supplementary Table 8

Pathway A	Pathway B	MuXTalk_score	Counts	in_XTalkDB_Gold
Apoptosis	Estrogen signaling pathway	2018.83733	120	B->A
Neurotrophin signaling pathway	TGF-beta signaling pathway	2015.85896	24	B->A
Apoptosis	TGF-beta signaling pathway	2014.77281	195	B->A
mTOR signaling pathway	TGF-beta signaling pathway	1022.12982	30	B->A
mTOR signaling pathway	Estrogen signaling pathway	1016.5687	54	B->A
Apoptosis	Hippo signaling pathway	1014.45492	44	B->A
Apoptosis	Wnt signaling pathway	1014.30758	169	B->A
Wnt signaling pathway	Prolactin signaling pathway	1014.07395	0	Neither
Apoptosis	Melanogenesis	1013.22843	2	B->A
Melanogenesis	Prolactin signaling pathway	1013.22175	0	Neither
Prolactin signaling pathway	Melanogenesis	1013.10225	0	Neither
TNF signaling pathway	Melanogenesis	1013.02262	0	B->A
VEGF signaling pathway	Estrogen signaling pathway	1012.83946	15	B->A
Toll-like receptor signaling pathway	ErbB signaling pathway	1012.82627	3	Neither
Neurotrophin signaling pathway	Estrogen signaling pathway	1012.48041	20	B->A
Prolactin signaling pathway	Wnt signaling pathway	1012.11132	0	Neither
Adherens junction	TNF signaling pathway	1011.56673	2	B->A
ErbB signaling pathway	Toll-like receptor signaling pathway	1009.9889	3	Neither
Insulin secretion	TGF-beta signaling pathway	1009.64181	58	Neither
JAK-STAT signaling pathway	TGF-beta signaling pathway	1009.15545	22	B->A
Adherens junction	Prolactin signaling pathway	1008.45344	0	Neither
Prolactin signaling pathway	Adherens junction	1007.98306	0	Neither
Melanogenesis	TGF-beta signaling pathway	1006.98922	1	B->A
Apoptosis	Insulin secretion	1006.76188	124	B->A
TGF-beta signaling pathway	Insulin secretion	1006.34269	58	Neither
Adherens junction	VEGF signaling pathway	1005.26494	0	B->A
Prolactin signaling pathway	Hippo signaling pathway	1004.68472	0	Neither
Melanogenesis	GnRH signaling pathway	1004.61921	0	Neither
Adherens junction	HIF-1 signaling pathway	1004.28279	0	B->A
Hippo signaling pathway	Prolactin signaling pathway	1004.26348	0	Neither
