# Supplementary figures and images for: Detecting and dissecting signaling crosstalk via the multilayer network integration of signaling and regulatory interactions
Source: Nucleic Acids Res. 2023 Nov 11;52(1):e5. doi: 10.1093/nar/gkad1035 (PMC10783515; doi:10.1093/nar/gkad1035)

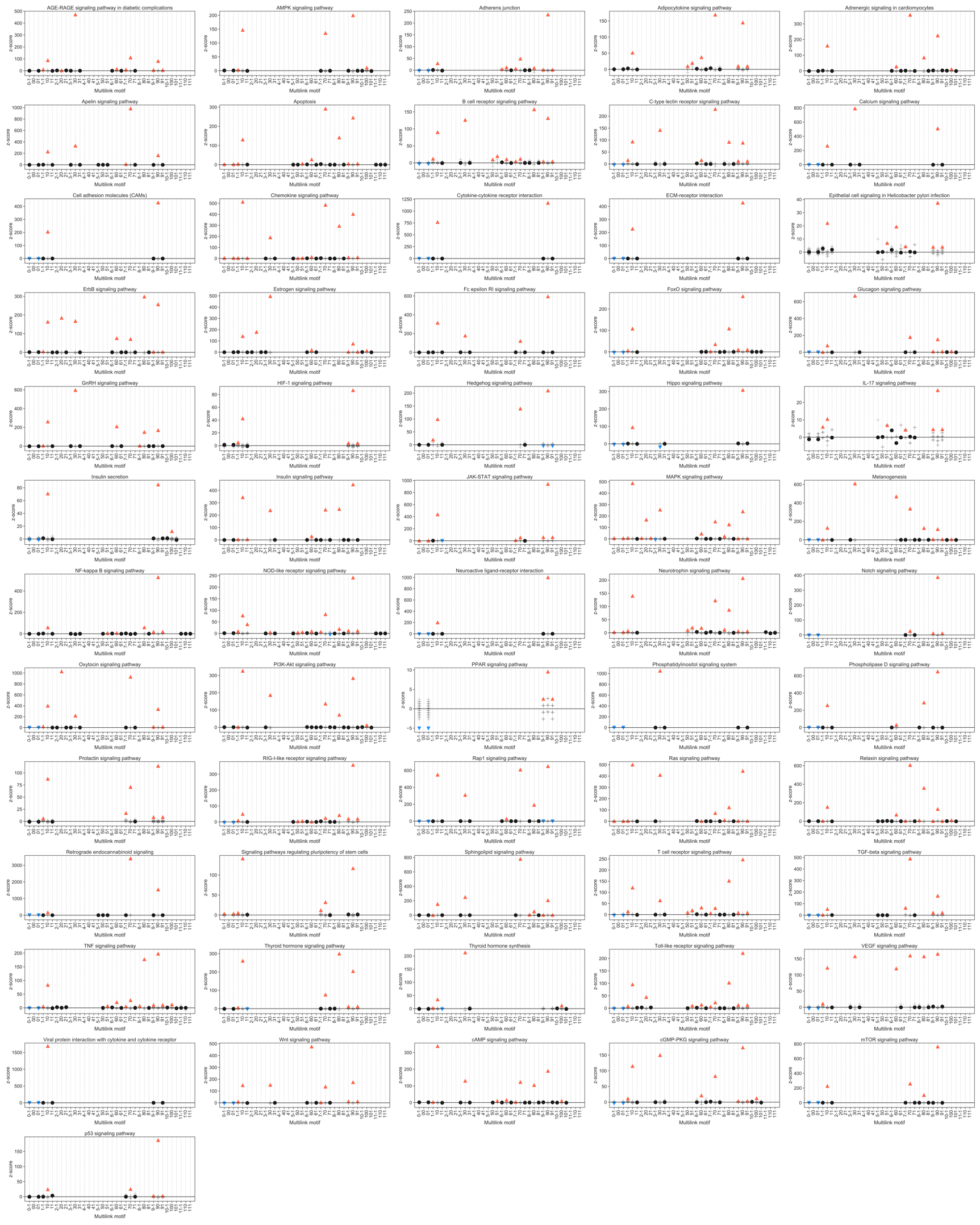

Supplement: gkad1035_Supplemental_Files [file gkad1035_supplemental_files.zip › Halu_MuXTalk_Supp_Fig5_p1e4.pdf]

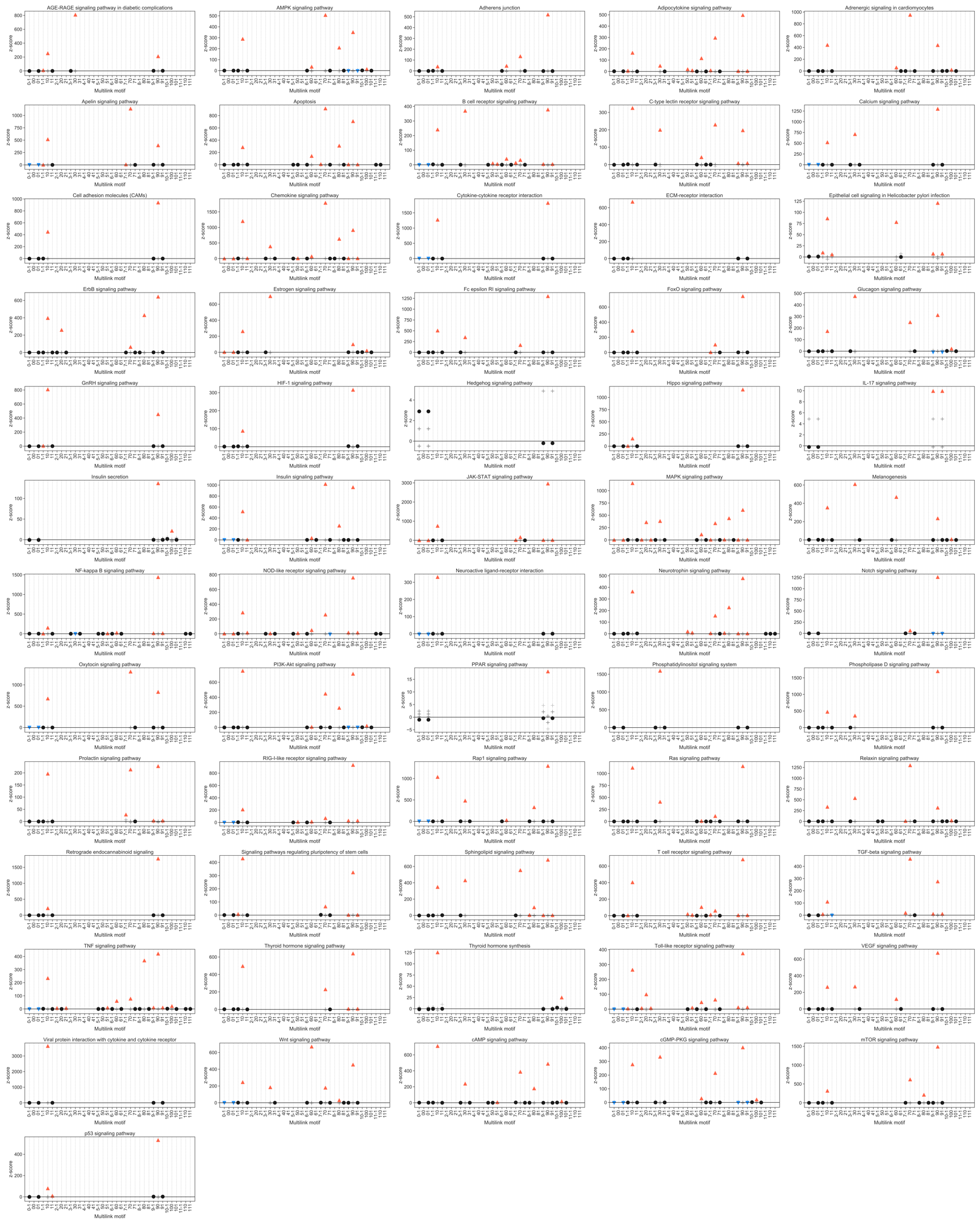

Supplement: gkad1035_Supplemental_Files [file gkad1035_supplemental_files.zip › Halu_MuXTalk_Supp_Fig6_p1e5.pdf]

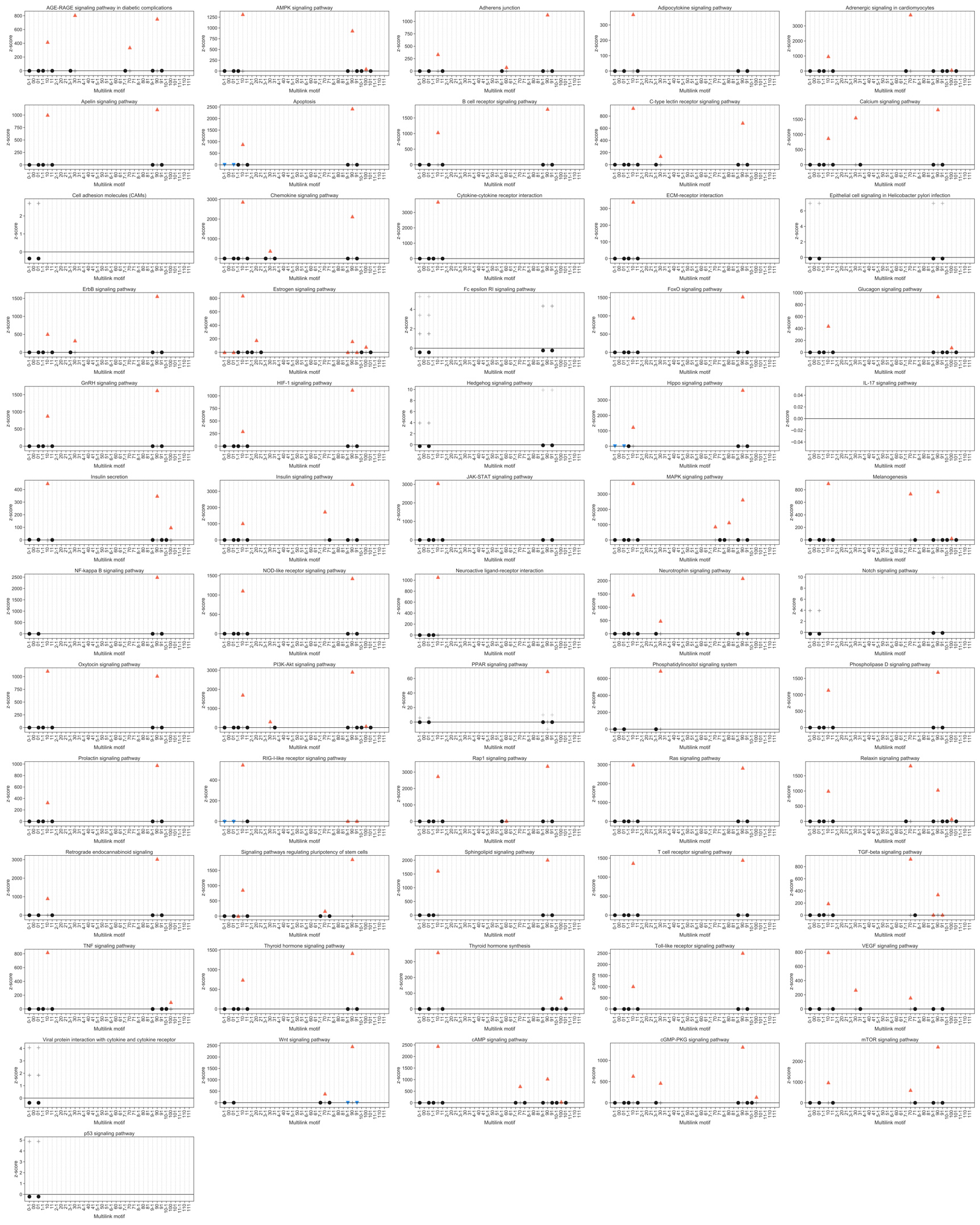

Supplement: gkad1035_Supplemental_Files [file gkad1035_supplemental_files.zip › Halu_MuXTalk_Supp_Fig7_p1e6.pdf]
